# Bifunctional immune checkpoint-targeted antibody-ligand traps that simultaneously disable TGFβ enhance the efficacy of cancer immunotherapy

**DOI:** 10.1038/s41467-017-02696-6

**Published:** 2018-02-21

**Authors:** Rajani Ravi, Kimberly A. Noonan, Vui Pham, Rishi Bedi, Alex Zhavoronkov, Ivan V. Ozerov, Eugene Makarev, Artem V. Artemov, Piotr T. Wysocki, Ranee Mehra, Sridhar Nimmagadda, Luigi Marchionni, David Sidransky, Ivan M. Borrello, Evgeny Izumchenko, Atul Bedi

**Affiliations:** 1 Department of Otolaryngology–Head and Neck Cancer Research, Johns Hopkins University School of Medicine, Baltimore, MD 21231 USA; 2 Sidney Kimmel Comprehensive Cancer Center, Johns Hopkins University School of Medicine, Baltimore, MD 21231 USA; 3 Department of Computer Science, Stanford University, Palo Alto, CA 94305 USA; 4 Insilico Medicine, Inc., Emerging Technology Centers, Johns Hopkins University at Eastern, B301, 1101 33rd Street, Baltimore, MD 21218 USA; 5 Department of Oncology, Johns Hopkins University School of Medicine, Baltimore, MD 21231 USA; 6 Department of Radiology and Radiological Science, Johns Hopkins Medical Institutions, Baltimore, MD 21287 USA; 7 Center for Computational Genomics, Johns Hopkins University School of Medicine, Baltimore, MD 21231 USA

## Abstract

A majority of cancers fail to respond to immunotherapy with antibodies targeting immune checkpoints, such as cytotoxic T-lymphocyte antigen-4 (CTLA-4) or programmed death-1 (PD-1)/PD-1 ligand (PD-L1). Cancers frequently express transforming growth factor-β (TGFβ), which drives immune dysfunction in the tumor microenvironment by inducing regulatory T cells (Tregs) and inhibiting CD8^+^ and T_H_1 cells. To address this therapeutic challenge, we invent bifunctional antibody–ligand traps (Y-traps) comprising an antibody targeting CTLA-4 or PD-L1 fused to a TGFβ receptor II ectodomain sequence that simultaneously disables autocrine/paracrine TGFβ in the target cell microenvironment (*a*-CTLA4-TGFβRII*ecd* and *a*-PDL1-TGFβRII*ecd*). *a*-CTLA4-TGFβRII*ecd* is more effective in reducing tumor-infiltrating Tregs and inhibiting tumor progression compared with CTLA-4 antibody (Ipilimumab). Likewise, *a*-PDL1-TGFβRII*ecd* exhibits superior antitumor efficacy compared with PD-L1 antibodies (Atezolizumab or Avelumab). Our data demonstrate that Y-traps counteract TGFβ-mediated differentiation of Tregs and immune tolerance, thereby providing a potentially more effective immunotherapeutic strategy against cancers that are resistant to current immune checkpoint inhibitors.

## Abstract (web summary)

Antitumor T cells can be inhibited by a TGFβ rich tumor microenvironment. The authors develop bifunctional proteins comprising CTLA-4 or PD-L1 immune checkpoint-targeted antibodies fused to a “TGFβ trap” and show that they counteract tumor immune tolerance and enhance the efficacy of these antibodies.

## Introduction

Genetic mutations accruing from the inherent genomic instability of tumor cells present neo-antigens that are recognized by the immune system. Cross-presentation of tumor antigens at the immune synapse between antigen-presenting dendritic cells and T lymphocytes can potentially activate an adaptive antitumor immune response that is mediated by CD4^+^ T-helper cells (T_H_1) and CD8^+^ cytotoxic effector cells, and sustained by tumor-reactive central memory T cells^1^. However, tumors continuously evolve to counteract and ultimately defeat such immune surveillance by co-opting and amplifying mechanisms of immune tolerance to evade elimination by the immune system^1–3^. This prerequisite for tumor progression is enabled by the ability of cancers to produce immunomodulatory factors that create a tolerogenic immune cell microenvironment^3^.

Transforming growth factor-β (TGFβ) is a multifunctional cytokine that is overexpressed in a majority of cancers^4^. The high-affinity binding of TGFβ to TGFβ receptor II (TGFβRII) recruits TGFβ receptor I into a heterotetrameric complex that initiates SMAD-mediated transcriptional activation or repression of several genes that control cell growth, differentiation, and migration^5^. Besides promoting epithelial-to-mesenchymal transition, invasion, and metastases of tumor cells, TGFβ has a critical role in regulating the adaptive immune system^6–9^. TGFβ suppresses the expression of interferon-γ (IFN-γ), restricts the differentiation of T_H_1 cells, attenuates the activation and cytotoxic function of CD8^+^ effector cells, and inhibits the development of central memory T cells^8–11^. Most significantly, TGFβ induces the differentiation of regulatory T cells (Tregs), a sub-population of immunosuppressive CD4^+^ T cells that express the interleukin-2 α-chain (CD25) and the forkhead box P3 (FOXP3) transcription factor^12–18^. TGFβ induces the expression of FOXP3, the signature transcription factor that determines and maintains the functional program of the Treg lineage^19–23^. FOXP3, in turn, induces the expression of cytotoxic T lymphocyte antigen-4 (CTLA-4), an immune-inhibitory receptor that restrains co-stimulation of T cells, and Galectin-9 (GAL-9), a ligand that engages the T-cell immunoglobulin domain and mucin domain-3 (TIM-3) immune-inhibitory receptor, and triggers exhaustion or apoptosis of effector T cells^24–28^. GAL-9 further interacts with TGFβ receptors to drive FOXP3 expression in a positive-feed forward autocrine loop involving SMAD3 activation to induce and maintain Tregs^29^. This ability of TGFβ to skew the differentiation of CD4^+^ T cells away from a T_H_1 phenotype toward a Treg lineage has significant clinical implications, as the functional orientation of tumor-infiltrating immune cells has a major impact on the outcome of patients with cancer^30^. Whereas T_H_1 cells, cytotoxic CD8^+^ T cells and central memory T cells are uniformly and strongly associated with a longer disease-free survival, infiltration of tumors with Tregs has been correlated with a poor prognosis in patients with several types of cancer^30–35^.

Current clinical efforts to counteract tumor-induced immune tolerance are focused on monoclonal antibodies, which counteract T-cell inhibitory receptors that function as immune checkpoints, such as CTLA-4 or programmed death-1 (PD-1)/PD-1 ligand (PD-L1)^36–41^. The CTLA-4 blocking antibody (Ipilimumab), two PD-1 antagonists (Pembrolizumab and Nivolumab), and three PD-L1 inhibitors (Atezolizumab, Avelumab, and Durvalumab) are currently approved in specific clinical indications for immunotherapy of cancers, such as melanoma, non-small cell lung cancer, head and neck cancer, or bladder cancer. Although a subset of patients with advanced cancers experience durable remissions and prolonged survival in response to CTLA-4 or PD-1/PD-L1 checkpoint inhibitors, the majority of patients do not respond to such therapy^42,43^.

A potential limitation of T-cell co-stimulation by current immune checkpoint inhibitors is a tumor milieu enriched with TGFβ, which strongly correlated with FOXP3 expression in our analysis of The Cancer Genome Atlas (TCGA) data set of diverse human cancers, including melanoma and breast cancer. We hypothesized that autocrine and paracrine TGFβ signaling in the localized microenvironment of tumor-infiltrating T cells could skew them toward Tregs and attenuate the activation of T_H_1 and CD8^+^ immune effector cells, thereby limiting the therapeutic efficacy of CTLA-4 or PD-1/PD-L1 antagonists^44,45^. As Tregs express and employ TGFβ and Gal-9 to maintain their own phenotype and function, enhancing the efficacy of immune checkpoint inhibitors requires a strategy to specifically break this hyperactive autocrine loop in tumor-infiltrating Tregs. To test this hypothesis and address this therapeutic challenge, we invented bifunctional antibody-ligand traps (Y-traps) comprising an antibody targeting either CTLA-4 or PD-L1, which is fused at the C terminus of the heavy chain (HC) to a TGFβRII ectodomain sequence to sequester and disable autocrine/paracrine TGFβ in the target cell microenvironment (*a*-CTLA4-TGFβRII*ecd* and *a*-PDL1-TGFβRII*ecd*)^46,47–49^. We find that *a*-CTLA4-TGFβRII*ecd* is significantly more effective in reducing and counteracting tumor-infiltrating Tregs, activating antitumor immunity, and inhibiting tumor progression compared with the CTLA-4 antibody, Ipilimumab. Likewise, *a*-PDL1-TGFβRII*ecd* exhibits superior antitumor efficacy compared with PD-L1 antibodies (Atezolizumab or Avelumab). Our data demonstrate that Y-traps simultaneously disable immune checkpoints and counteract TGFβ-mediated differentiation of Tregs and immune tolerance, thereby providing a more effective immunotherapeutic strategy against cancers that fail to respond to current immune checkpoint inhibitors.

## Results

### TGFβ signaling correlates with *FOXP3* expression in cancers

**Fig. 1:**
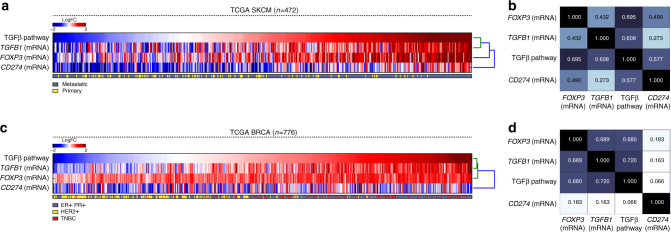
TGFβ pathway activation correlates with elevated *TGFB1* and *FOXP3* mRNA expression in TCGA-SKCM and TCGA-BRCA data sets. iPANDA software suite for analysis of intracellular signaling pathway activation based on transcriptomic data was used to estimate the level of TGFβ signaling in melanoma or breast samples from TCGA-SKCM (*n* = 472) and TCGA-BRCA data set, respectively (*n* = 776). TCGA transcriptomic data from normal breast tissue (*n* = 114) or skin biopsy of healthy women (*n* = 122) was used as a reference after proper normalization. The TGFβ pathway activation scores for **a** 472 melanomas (metastatic and primary) and **c** 776 breast tumors (ER/PR positive, HER2 positive, and TNBC) are sorted from low to high. The corresponding expression of *TGFB1* gene, *FOXP3*, and *CD274* is shown for each patient. The units on the color bar represent the pathway activation score or the fold change in the gene expression on the logarithmic scale in comparison with the average normal control level. Correlation matrix for melanoma (**b**) and breast cancer (**d**) are shown (numbers in plot indicate correlation coefficient value). Pairwise correlations between TGFβ pathway activation and message for *TGFB1*, *FOXP3*, and *CD274* were computed using the Pearson's correlation coefficient. The *p*-values were calculated with the null hypothesis that the tested samples are unrelated. The *p*-values for all correlation < 0.05

We used iPANDA, a bioinformatics software suite for qualitative analysis of intracellular signaling pathway activation based on transcriptomic data^50,51^, to assess the level of TGFβ signaling in TCGA data sets of different types of cancer and investigate whether the TGFβ pathway activation in tumors is correlated with the level of expression of *FOXP3*, the signature transcription factor of the Treg lineage. Analysis of transcriptomic data from a skin cutaneous melanoma (SKCM) data set (*n* = 472), using skin biopsy of healthy women (*n* = 122) as a reference, showed that upregulation in TGFβ signaling strongly correlated with increased messenger RNA expression levels of *TGFB1* and *FOXP3* (Fig. 1a, b). The strong correlation between TGFβ pathway activation and *FOXP3* expression was also noted in a TCGA breast cancer data set (*n* = 776), using normal breast tissue as a reference (Fig. 1c, d). Among breast cancers, TGFβ pathway activation and corresponding elevation of *FOXP3* was especially striking in triple-negative breast cancer (TNBC) (Fig. 1c), an aggressive subtype that lacks expression of hormone receptors (estrogen receptor (ER)/progesterone receptor (PR)) and HER2/neu, and has a higher risk of metastases and death within 5 years of diagnosis. Although TGFβ and PD-L1 can cooperate to induce expression of FOXP3, expression of *PD-L1 (CD274)* mRNA did not exhibit a corresponding or consistent correlation with *FOXP3* mRNA expression (Fig. 1d). The strong correlation of TGFβ activation with *FOXP3* expression supports a crucial role of autocrine/paracrine TGFβ signaling in induction and maintenance of Tregs in diverse cancers.

### Design and bifunctional target binding of *a*-CTLA4-TGFβRII

**Fig. 2:**
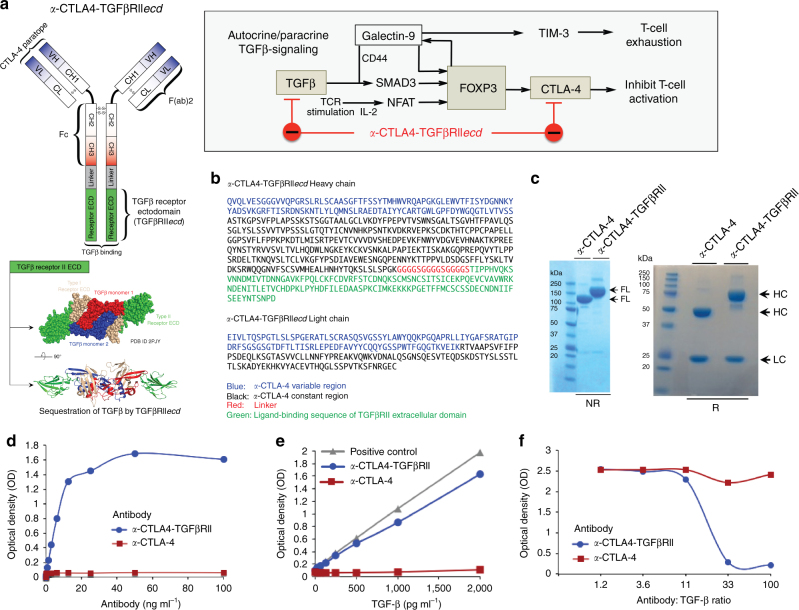
Design and bifunctional target binding ability of *a*-CTLA4-TGFβRII. **a** Autocrine/paracrine TGFβ-induced expression of FOXP3, the signature transcription factor of the Treg lineage. FOXP3 induces the expression of CTLA-4 and Galectin-9, a ligand that engages TIM-3 and triggers exhaustion or apoptosis of effector T cells. Galectin-9 further interacts with TGFβ receptors to drive FOXP3 expression in a positive-feed forward autocrine loop involving SMAD3 activation to induce and maintain Tregs. Schematic representation of the structure and targets of *a*-CTLA4-TGFβRII are shown. *a*-CTLA4-TGFβRII was designed by fusing the C terminus of the heavy chain of a human *a*-CTLA-4 antibody with a ligand-binding sequence of the extracellular domain of TGFβ Receptor II (TGFβRII ECD) via a flexible linker peptide, (GGGGS)_3_. **b** Amino acid sequences of the heavy chain and light chain of *a*-CTLA4-TGFβRII. **c** SDS-PAGE (unreduced-NR and reduced-R) comparing the molecular weight of *a*-CTLA4-TGFβRII and *a*-CTLA-4 antibody (FL, full-length; HC, heavy chain; LC, light chain). **d**,**e** Bifunctional ability of *a*-CTLA4-TGFβRII to simultaneously bind CTLA-4 and TGFβ1 using a ‘double-sandwich’ ELISA, wherein *a*-CTLA4-TGFβRII or *a*-CTLA-4 antibody was added to CTLA-4-Fc-coated plates, followed by rhTGFβ1 that was detected by a biotinylated anti-human TGFβ1 antibody. TGFβRII-Fc-coated plates were used as a TGFβ-binding positive control. **f** Standard ELISA showing the ability of *a*-CTLA4-TGFβRII to compete with a TGFβ capture antibody for binding to TGFβ1. For **d**–**f**, the data show the optical density (OD) values (mean of three replicate wells for each assay condition) from a representative of two independent experiments

Anti-CTLA4-TGFβRII*ecd* (*a*-CTLA4-TGFβRII) was designed to simultaneously target both CTLA-4 and TGFβ by fusing the C terminus of the HC of a human anti-CTLA-4 antibody with a ligand-binding sequence of the extracellular domain of TGFβRII via a flexible linker peptide (Fig. 2a, b). Protein identification of the purified antibody from CHO-K1 cell supernatants was performed by liquid chromatography Fourier transform tandem mass spectrometry (LC–MS/MS) to confirm the amino acid sequence of the HC of *a*-CTLA4-TGFβRII (Fig. 2b). SDS-polyacrylamide gel electrophoresis (PAGE) under reducing (R) and non-reducing (NR) conditions was used to compare the full-length (FL), HC, and light chain (LC) of *a*-CTLA4-TGFβRII and *a*-CTLA-4 antibody (Fig. 2c). MS analysis confirmed the expected higher molecular weight of the HC of *a*-CTLA4-TGFβRII (65.697 kDa) compared with the HC of *a*-CTLA-4 antibody (49.256 kDa). The bifunctional ability of *a*-CTLA4-TGFβRII to simultaneously bind CTLA-4 and TGFβ1 was confirmed by enzyme-linked immunosorbent assay (ELISA), wherein *a*-CTLA4-TGFβRII was added to CTLA-4-Fc-coated plates, followed by recombinant human TGFβ (rhTGFβ1) that was detected by a biotinylated anti-human TGFβ1 antibody (Fig. 2d, e). Unlike *a*-CTLA-4, *a*-CTLA4-TGFβRII exhibited the additional ability to compete with a TGFβ capture antibody for binding to TGFβ1 (Fig. 2f).

### *a*-CTLA4-TGFβRII counteracts Tregs and T_H_17 differentiation

**Fig. 3:**
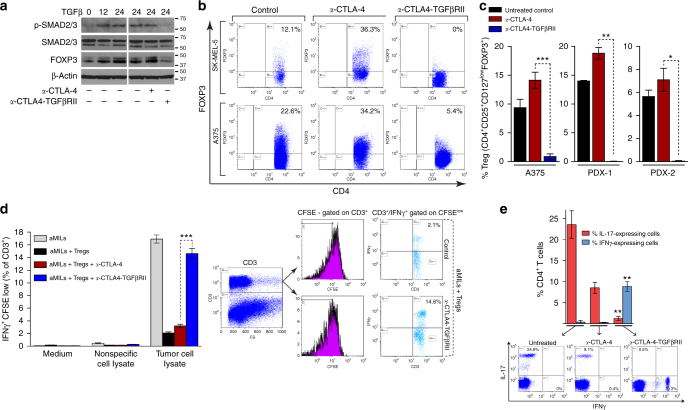
*a*-CTLA4-TGFβRII counteracts tumor-infiltrating Tregs and inhibits T_H_17 differentiation. **a** Immunoblot analyses of the effect of *a*-CTLA4-TGFβRII or *a*-CTLA-4 on TGFβ-induced SMAD-2/3 phosphorylation and expression of FOXP3 in human PBMC. PBMCs were cultured for 24–48 h with rhIL-2 (100 IU ml^−1^) and anti-CD3/anti-CD28-coated Dynabeads in the presence or absence of rhTGFβ1 (2.5 ng ml^−1^) with or without either *a*-CTLA4-TGFβRII or *a*-CTLA-4 (5 μg ml^−1^). **b**, **c** Flow cytometric analysis of the in vivo effect of *a*-CTLA4-TGFβRII or *a*-CTLA-4 on bone marrow (BM) and tumor-infiltrating Tregs in human melanoma tumor-bearing NSG mice that were immune reconstituted with tumor-matched HLA A2^+^ human CD34^+^ bone marrow cells. Mice engrafted with human CD3^+^ cells were inoculated subcutaneously with either human melanoma tumor cells (A375 or SK-MEL-5) or patient-derived melanoma tumor xenografts (PDX-1 and PDX-2). Tumor-bearing mice (A375; SK-MEL-5; PDX-1; PDX-2) were randomized into groups and treated with either *a*-CTLA4-TGFβRII or *a*-CTLA-4 (5 mg kg^−1^ i.p. weekly), or vehicle alone (untreated control). **b** Representative flow data of FOXP3 expression in BM CD4^+^CD25^+^ T cells in A375 or SK-MEL-5 tumor-bearing mice from each treatment or control group. **c** The percentage of Tregs (CD4^+^/CD25^high^/CD127^low^/FOXP3^+^) in tumor-infiltrating lymphocytes in A375- or PDX-bearing mice (five animals per treatment group for A375 and four mice for PDXs). **d** Effect of *a*-CTLA4-TGFβRII or *a*-CTLA-4 on the suppression of tumor-reactive T cells by autologous Tregs in tumor-infiltrated BM from a patient. Marrow-infiltrating lymphocytes (MILs) stimulated with anti-CD3/anti-CD28-beads and rhIL-2 were carboxyfluorescein N-hydroxysuccinimidyl ester (CFSE) labeled and added to autologous BM that had been pulsed with either myeloma cell lysate (tumor-specific antigen) or nonspecific antigen in the presence or absence of autologous CD4^+^/CD25^+^ MILs, with or without either *a*-CTLA4-TGFβRII or *a*-CTLA-4 (5 μg ml^−1^) for 3 days. Tumor antigen-reactive T cells (CD3^+^/CFSE^low^/IFNγ^+^) were quantified by flow cytometry (three in vitro replicates for each experimental group). **e** PBMCs were stimulated with anti-CD3/CD28 beads in the presence of IL17-skewing cytokines (10 ng ml^−1^ IL-6, 5 ng ml^−1^ TGFβ, 10 ng ml^−1^ anti–IFN-γ, and 10 ng ml^−1^ anti–IL-4) with or without either *a*-CTLA4-TGFβRII or *a*-CTLA-4 (5 μg ml^−1^) for 3 days. Following addition of Leukocyte Activation Cocktail (2 μl ml^−1^ for 4 h), cells were analyzed by flow cytometry for intracellular expression of IL-17 and IFN-γ in CD4 + T cells (three in vitro replicates for each experimental group). Asterisks represent statistical significance between the *a*-CTLA4-TGFβRII and *a*-CTLA-4 treatments. Representative flow cytometric dot plots are shown

The FOXP3 transcription factor governs the differentiation and function of Tregs. The transcription factors SMAD3 and nuclear factor of activated T-cells (NFAT) are required for activation of a *FOXP3* enhancer, and both factors are essential for induction of FOXP3 in primary T cells. TGFβ-activated SMAD-2/3 cooperates with interleukin (IL)-2-activated NFAT to induce FOXP3 expression and promote the conversion of naïve CD4^+^ T cells to FOXP3-expressing Treg cells (induced Tregs or iTregs) that mediate immune tolerance^19^ (Fig. 2a). Consistent with these observations, treatment with rhTGFβ1 induced the phosphorylation of SMAD-2/3 and increased expression of FOXP3 in human peripheral blood mononuclear cells (PBMCs) costimulated with anti-CD3/anti-CD28-coated beads and rhIL-2 (Fig. 3a, left panel).

*a*-CTLA4-TGFβRII is designed to exploit the FOXP3-mediated expression of CTLA-4 on Tregs to decorate the targeted cells with a decoy TGFβRII ectodomain that captures and disables TGFβ in their localized microenvironment (Fig. 2a, b). We examined the ability of *a*-CTLA4-TGFβRII to inhibit TGFβ-induced SMAD-2/3 phosphorylation and expression of FOXP3 in human T cells. Human PBMC were stimulated with rhIL-2 and anti-CD3/anti-CD28-coated beads in the presence of rhTGFβ1 with or without either *a*-CTLA4-TGFβRII or *a*-CTLA-4. Unlike *a*-CTLA-4, *a*-CTLA4-TGFβRII counteracted TGFβ-induced SMAD-2/3 phosphorylation and FOXP3 expression in co-stimulated T cells (Fig. 3a, right panel).

The in vivo effect of *a*-CTLA4-TGFβRII on Tregs was examined in human melanoma tumor-bearing NSG mice (NOD/Shi-*scid IL-2rg*^*null*^) that were immune reconstituted with tumor-matched human leukocyte antigen (HLA) A2 + human CD34^+^ bone marrow (BM) cells. The production of TGFβ by A375 tumor cells (842 pg per 10^6^ cells per 24 h) and SK-MEL-5 tumor cells (513 pg per 10^6^ cells per 24 h) was confirmed by ELISA assay. Tregs in BM and tumor-infiltrating T cells isolated from tumor-bearing mice treated with either *a*-CTLA4-TGFβRII or *a*-CTLA-4 and their untreated counterparts were measured by immunophenotype analysis. Compared with *a*-CTLA-4, treatment of tumor-bearing mice with *a-*CTLA4-TGFβRII resulted in a marked decline of FOXP3-expression in CD4^+^ cells (Fig. 3b). A375 or patient-derived tumor xenograft (PDX)-bearing mice treated with *a*-CTLA4-TGFβRII exhibited a significant reduction of tumor-infiltrating Tregs (CD4^+^CD25^+^CD127^low^FOXP3^+^ cells) compared to those treated with *a*-CTLA-4 (*p* < 0.001 for A375, *p* < 0.003 for PDX1, and *p* < 0.02 for PDX2; Student’s unpaired *t*-test) (Fig. 3c). These data show that *a*-CTLA4-TGFβRII counteracts FOXP3^+^ Treg specification in a TGFβ-enriched tumor microenvironment.

As FOXP3 is instrumental for the suppressive function of Tregs, the relative ability of *a*-CTLA4-TGFβRII and *a*-CTLA-4 to counteract Treg-mediated suppression of tumor-reactive T cells was also examined using tumor-infiltrated BM from a patient. Anti-CD3/anti-CD28 and rhIL-2 activated CD3^+^ marrow-infiltrating lymphocytes (aMILs)^52,53^ were CFSE labeled and added to autologous BM that had been pulsed with either tumor cell lysate (tumor-specific antigen) or nonspecific antigen in the presence or absence of autologous CD4^+^/CD25^+^ Tregs isolated from the same patient’s BM. Following culture of these cells for 3 days with or without either *a*-CTLA4-TGFβRII or *a*-CTLA-4, tumor antigen-reactive T cells (CD3^+^/CFSE^low^/IFNγ^+^) were quantified by immunophenotype analyses (Fig. 3d). As expected, the addition of autologous Tregs suppressed the activation of tumor antigen-reactive T cells (CD3^+^/CFSE^low^/IFNγ^+^) in anti-CD3/anti-CD28-activated aMILs stimulated with tumor antigen-pulsed autologous BM. *a*-CTLA4-TGFβRII was far more effective than *a*-CTLA-4 in counteracting Treg-mediated suppression and restoring activation of tumor antigen-specific T cells in the presence of autologous Tregs (Fig. 3d). These data demonstrate that *a*-CTLA4-TGFβRII is more effective than *a*-CTLA-4 in counteracting Tregs in the tumor microenvironment.

The differentiation of CD4^+^ T cells into T_H_1, T_H_17, or iTreg cell lineages is determined by the cytokine milieu^54^. Whereas IFN-γ drives T_H_1 differentiation, TGFβ is required for differentiation of both iTreg and T_H_17 cells. Although TGFβ cooperates with IL-2 to induce iTreg differentiation^13,55,56^, TGFβ promotes T_H_17 differentiation in the presence of proinflammatory cytokines, such as IL-6^57–61^. In contrast to T_H_1 cells that are strongly associated with good clinical prognosis for all cancer types, T_H_17 cells are associated with tumor-promoting inflammation and autoimmune pathology^30,62–64^. As *a*-CTLA4-TGFβRII can render the targeted T cells incapable of responding to TGFβ signals in their immediate milieu, we examined whether *a*-CTLA4-TGFβRII also skews the differentiation of CD4^+^ T cells away from T_H_17 cells toward an IFN-γ-expressing T_H_1 phenotype. Whereas the T_H_17 phenotype of CD4^+^ T cells costimulated with anti-CD3/anti-CD28 beads under T_H_17 skewing conditions was maintained in the presence of *a*-CTLA-4, *a*-CTLA4-TGFβRII was able to abrogate the expression of IL-17 in CD4^+^ T cells and switch them to an IFN-γ-expressing T_H_1 phenotype (Fig. 3e).

### Superior antitumor immunity and efficacy of *a*-CTLA4-TGFβRII

**Fig. 4:**
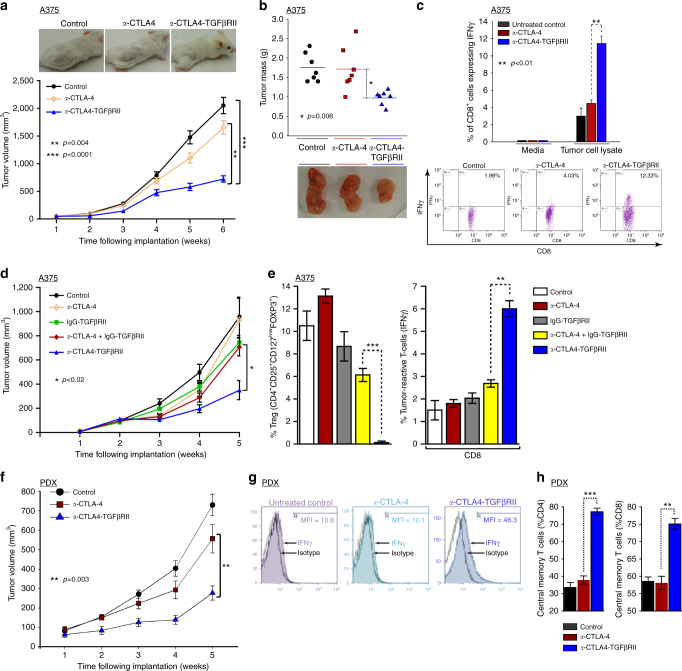
*a*-CTLA4-TGFβRII exhibits superior antitumor efficacy with elevation tumor-reactive IFNγ-expressing and central memory T cells. Effect of the indicated treatments on melanoma tumor xenografts in NSG mice that were immune reconstituted with tumor-matched HLA A2^+^ human CD34^+^ BM cells. Mice engrafted with human CD3^+^ cells were inoculated subcutaneously with either human melanoma tumor cells (A375) or patient-derived melanoma tumor xenografts. At 10d following tumor inoculation, mice were randomized into groups and treated with the indicated antibodies (5 mg kg^−1^ i.p. weekly) or vehicle alone (untreated control). **a** Representative images and in vivo tumor growth curves in A375 tumor-bearing mice: (mean + SEM of 12 mice in each indicated group). **b** Representative images and mass of tumors at the end of treatment in A375 tumor-bearing mice: (seven mice in each indicated group). **c** Flow cytometric analysis of the comparative in vivo effect of the indicated treatments on tumor-reactive IFN-γ expression in CD8^+^ T cells in A375 tumor-bearing mice. Representative flow data is shown (mean ± SEM). **d**, **e** Comparative antitumor efficacy of *a*-CTLA4-TGFβRII, *a*-CTLA-4, nonspecific IgG-TGFβRII, and the combination of *a*-CTLA-4 and IgG-TGFβRII in A375 tumor-bearing mice. **d** Tumor growth curves (mean ± SEM of five mice). The *p-*value (*p* < 0.02, Student’s unpaired *t*-test) denotes significant difference between *a*-CTLA4-TGFβRII and each other treatment group. **e** Flow cytometric analysis of infiltrating FOXP3-expressing Tregs (left) and tumor-reactive IFN-γ expressing CD8^+^ T cells (right). **f** Tumor growth curves in melanoma PDX-bearing mice: (mean ± SEM of five mice). **g** Representative flow data of in vivo effect of *a*-CTLA4-TGFβRII and *a*-CTLA-4 on tumor-specific IFN-γ expression in CD8^+^ T cells in PDX-bearing mice. **h** Flow cytometric analysis of the comparative in vivo effect of *a*-CTLA4-TGFβRII and *a*-CTLA-4 on tumor-infiltrating central memory T cells (CD45RO^high^CD62L^high^) in PDX-bearing mice

As *a*-CTLA4-TGFβRII effectively counteracted tumor-infiltrating Tregs in vivo, we examined its ability to increase tumor-reactive IFNγ expression in T cells and inhibit tumor growth in human melanoma tumor-bearing NSG mice that were immune reconstituted with matched HLA A2^+^ human BM CD34^+^ cells. Treatment with *a*-CTLA4-TGFβRII was significantly more effective at inhibiting the growth of A375 tumors compared with *a*-CTLA-4 (*p* < 0.004, Student’s unpaired *t*-test) or untreated controls (*p* < 0.0001, Student’s unpaired *t*-test) (Fig. 4a, b). Consistent with its superior antitumor efficacy, treatment of A375-tumor-bearing mice with *a*-CTLA4-TGFβRII resulted in a greater elevation in tumor-reactive IFN-γ-expressing CD8^+^ cells compared with treatment with *a*-CTLA-4 (Fig. 4c). We next evaluated the comparative antitumor efficacy of *a*-CTLA4-TGFβRII, nonspecific IgG-TGFβRII, and the combination of *a*-CTLA-4 and IgG-TGFβRII using the same model. Treatment of tumor-bearing mice with *a*-CTLA4-TGFβRII was significantly more effective at inhibiting tumor progression (*p* < 0.02, Student’s unpaired *t*-test) (Fig. 4d), reducing FOXP3^+^ expressing Tregs (*p* < 0.001, Student’s unpaired *t*-test) (Fig. 4e, left) and elevating tumor reactive IFNγ-expressing CD8^+^ cells (*p* < 0.01, Student’s unpaired *t*-test) (Fig. 4e, right) compared with the *a*-CTLA-4 alone, IgG-TGFβRII alone, and even the combination of *a*-CTLA-4 and nonspecific IgG-TGFβRII (Fig. 4d, e). Mice treated with *a*-CTLA4-TGFβRII maintained serum hepatic enzymes within a normal range of liver function (mean ± SEM) (alanine transaminase (14 ± 3 U l^−1^), aspartate transaminase (67 ± 4 U l^−1^), alkaline phosphatase (68.5 ± 6 U l^−1^), total bilirubin (0.25 ± 0.1 mg ml^−1^)) and demonstrated no loss of body weight during the course of experiment. The superior antitumor efficacy of *a*-CTLA4-TGFβRII compared with *a*-CTLA-4 was further confirmed in immune-reconstituted NSG mice bearing primary PDX (Fig. 4f). Accordingly, treatment with *a*-CTLA4-TGFβRII also resulted in higher tumor-reactive IFN-γ-expressing CD8^+^ cells compared to treatment with *a*-CTLA-4 in mice bearing human melanoma PDX (Fig. 4g). The comparative in vivo effect of *a*-CTLA4-TGFβRII and *a*-CTLA-4 on the differentiation of tumor-infiltrating T cells into central memory T cells (CD45RO^high^CD62L^high^) was also evaluated in tumors collected from PDX-bearing immune-reconstituted mice. Treatment with *a*-CTLA4-TGFβRII was more effective than *a*-CTLA-4 in increasing the percentage of CD4^+^ and CD8^+^ T cells with a central memory phenotype (Fig. 4h). These results demonstrate that TGFβ in the tumor microenvironment reduces tumor-reactive IFN-γ-expressing CD8^+^ cells and tumor-infiltrating central memory T cells, and that *a*-CTLA4-TGFβRII is required to effectively counteract these effects by rendering the targeted T cells incapable of responding to autocrine/paracrine TGFβ signals in their immediate milieu.

### *a*-CTLA4-TGFβRII is more effective than *a*-CTLA-4 and *a*-PD1

**Fig. 5:**
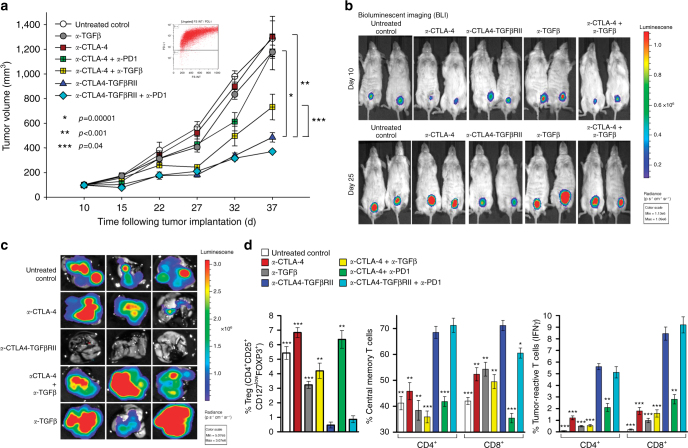
*a*-CTLA4-TGFβRII inhibits tumor growth more effectively than the combination of *a*-CTLA-4 and *a*-PD1. NSG mice immune reconstituted with tumor-matched HLA A2^+^ human CD34^+^ BM cells and bearing MDA-MB231-Luc human TNBC tumor xenografts were treated (5 mg kg^−1^ i.p. weekly) with vehicle alone (untreated control) or the following antibodies (either alone or in combination), as indicated: *a*-CTLA4-TGFβRII; *a*-CTLA-4; *a*-TGFβ (1D11); *a*-PD1 (Pembrolizumab); (five to six mice per group). **a** In vivo tumor growth curves (mean ± SEM). *p-*values were derived using unpaired, two-sided *t*-test. **b** Bioluminescence assay of primary tumors in untreated controls or the indicated treatment groups at 10 and 25 days after tumor cell inoculation. **c** Bioluminescence assay of lung metastases in untreated controls or the indicated treatment groups at 40 days post tumor inoculation. **d** Immunophenotype analysis of the effect of the indicated treatments on the percentage of Tregs (CD4^+^/CD25^high^/CD127^low^/FOXP3^+^), tumor-reactive IFN-γ-expressing CD8^+^ T cells, and central memory CD4^+^ and CD8^+^ T cells (CD45RO^high^CD62L^high^). Bars represent mean ± SEM of three animals per treatment group. Asterisks above each bar denote the statistical significance of the difference between the indicated group and *a*-CTLA4-TGFβRII (blue bar)

Besides CTLA-4, engagement of PD-1 by PD-L1 expressed on tumor cells or T cells also inhibits antitumor T cells. Although monoclonal antibodies (mAbs) against PD-1, such as Nivolumab or Pembrolizumab, are effective in some patients, the vast majority of cancers fail to respond to either PD-1 blockade or even dual checkpoint inhibition with *a*-CTLA-4 and *a*-PD-1. Therefore, we investigated the ability of *a*-CTLA4-TGFβRII to elicit antitumor immunity and inhibit the growth and metastases of cancers that are refractory to current checkpoint inhibitors, such as TNBC. Approximately 15–25% of patients with breast cancer have TNBC, an aggressive type that does not respond to hormonal agents or targeted therapy and has an increased risk of metastases. As TNBC is representative of a tumor type that exhibits a TGFβ/FOXP3 signature of Treg-mediated immune tolerance (Fig. 1c), we used human immune reconstituted NSG mice bearing the bioluminescent human MDA-MB-231-luc (D3H2LN) TNBC cell line that expresses elevated PD-L1 (Fig. 5a, inset) and TGFβ (531 pg per 10^6^ cells per 24 h) and exhibits enhanced lung metastases. Treatment of MDA-MB-231-luc-bearing mice with either *a*-CTLA-4 alone or the combination of *a*-CTLA-4 and *a*-PD-1 mAbs failed to inhibit tumor growth or lung metastases compared with untreated animals (Fig. 5a–c). In contrast, treatment with *a*-CTLA4-TGFβRII was significantly more effective at inhibiting the progression of MDA-MB-231-luc tumors compared with untreated controls (*p* < 0.00001, Student’s unpaired *t*-test), or animals treated with either *a*-CTLA-4 alone (*p* < 0.001, Student’s unpaired *t*-test) or the combination of *a*-CTLA-4 and *a*-PD-1 mAbs (*p* < 0.0001, Student’s unpaired *t*-test) (Fig. 5a, b). In addition, *a*-CTLA4-TGFβRII exhibited significantly better antitumor efficacy compared with either *a*-TGFβ (*p* < 0.001, Student’s unpaired *t*-test) or a combination of *a*-CTLA-4 and *a*-TGFβ (*p* < 0.04, Student’s unpaired *t*-test) (Fig. 5a, b), and was more effective in inhibiting lung metastases (Fig. 5c). Consistent with its superior antitumor efficacy, *a*-CTLA4-TGFβRII was more effective in reducing Tregs, elevating tumor-reactive IFN-γ-expressing CD8^+^ cells and increasing the CD4^+^ and CD8^+^ central memory T cells compared with the combination of *a*-CTLA-4 and *a*-PD-1 mAbs (Fig. 5d).

### Design and bifunctional target binding of *a*-PDL1-TGFβRII

PD-L1 is overexpressed on tumor cells as well as tumor-infiltrating T cells, where it cooperates with TGFβ to inhibit T-cell activation and induce and maintain immunosuppressive Treg cells. Although TGFβ and PD-L1 can cooperate to induce FOXP3, our analysis of both TCGA data sets showed that the correlation of TGFβ pathway activation with *FOXP3* expression was substantially stronger than its correlation with PD-L1 (*CD274*) mRNA (Fig. 1). These data suggest that PD-L1/PD-1 checkpoint and TGFβ signaling exercise independent, yet cooperative mechanisms of immune tolerance, thereby supporting a therapeutic rationale for simultaneously counteracting both axes in the tumor immune microenvironment.

**Fig. 6:**
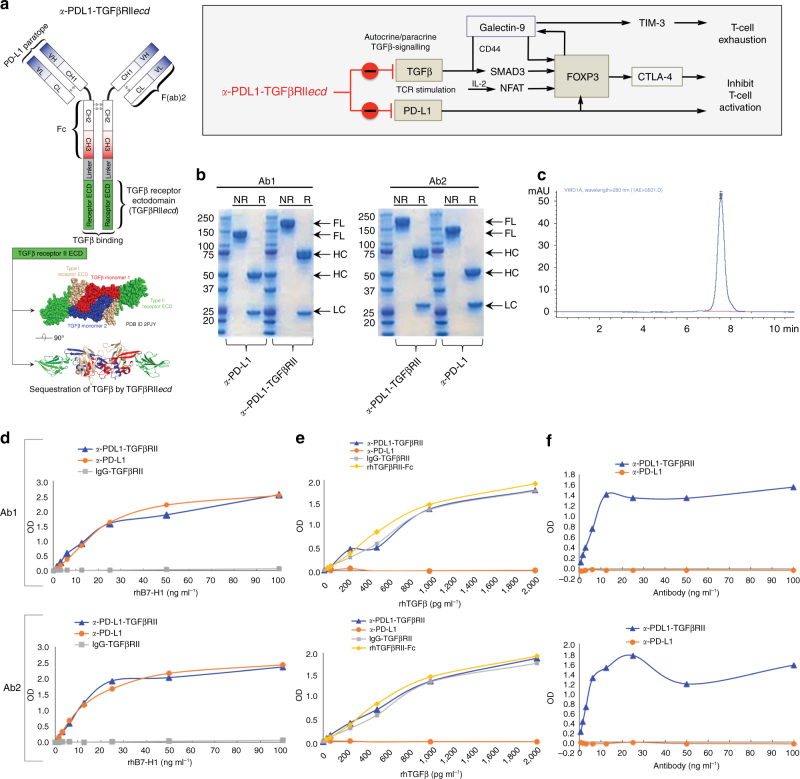
Design and bifunctional target binding ability of anti-PDL1-TGFβRII. **a** Schematic representation of PD-L1 and TGFβ entrained independent and cooperative mechanisms of immune tolerance. Schematic structure and targets of *a*-PDL1-TGFβRII are shown. *a*-PDL1-TGFβRII was designed by fusing the C terminus of the heavy chain of a human *a*-PD-L1 antibody with a ligand-binding sequence of the extracellular domain of TGFβ Receptor II (TGFβRII ECD) via a flexible linker peptide, (GGGGS)_3._ **b** SDS-PAGE under non-reducing (NR) and reducing (R) conditions was used to compare the full-length, heavy chain and light chain of *a*-PDL1-TGFβRII and *a*-PD-L1 antibody. Figure shows the results of SDS-PAGE analyses of each of two separate *a*-PDL1-TGFβRII Y-traps (Ab1 and Ab2) and their respective human *a*-PD-L1 antibody (atezolizumab and avelumab). **c** SEC-HPLC analysis of purified *a*-PDL1-TGFβRII; **d** ELISA showing the comparative ability of *a*-PDL1-TGFβRII and *a*-PD-L1 antibody to bind PD-L1. Biotinylated recombinant human PD-L1 (rh B7-H1-biotin; 0–100 ng ml^−1^) was added to plates coated with *a*-PDL1-TGFβRII or *a*-PD-L1 antibody (1 μg ml^−1^), followed by detection with HRP-Avidin. Plates coated with non-specific IgG-TGFβRII showed no binding to PD-L1 and served as a negative control to analyze the binding ability of the test samples. **e** ELISA showing the comparative ability of *a*-PDL1-TGFβRII and *a*-PD-L1 antibody to bind TGFβ. Recombinant human TGFβ (rhTGFβ1; 0–2,000 pg ml^−1^) was added to plates coated with *a*-PDL1-TGFβRII or *a*-PD-L1 antibody (1 μg ml^−1^), which was detected by biotinylated *a*-TGFβ1 and HRP-Avidin. Plates coated with nonspecific IgG-TGFβRII and rhTGFβRII-Fc served as positive controls to analyze the binding ability of the test samples to TGFβ. **f** ELISA showing the ability of *a*-PDL1-TGFβRII to simultaneously bind both PD-L1 and TGFβ. Anti-PDL1-TGFβRII or *a*-PD-L1 antibody (0–100 ng ml^−1^) was added to PD-L1-Fc coated plates (1 μg ml^−1^), followed by rhTGFβ1 (100 ng ml^−1^) that was detected by a biotinylated anti-human TGFβ1 antibody. For **d**–**f**, the data show the optical density (OD) values (mean of three replicate wells for each assay condition) from a representative of two independent experiments

Anti-PDL1-TGFβRII*ecd* (*a*-PDL1-TGFβRII) is a bifunctional antibody–ligand trap that was designed to target PD-L1 and simultaneously inactivate TGFβ by fusion of an extracellular domain sequence of TGFβRII to the C terminus of the HC of anti-PD-L1 antibody via a flexible linker sequence, (GGGGS)_3_ (Fig. 6a, b). SDS-PAGE of two different anti-PD-L1 antibodies (Atezolizumab and Avelumab) and their corresponding anti-PDL1-TGFβRII products (Ab1 and Ab2) under R and NR conditions showed the expected higher molecular weight of the HC of anti-PDL1-TGFβRII. Size exclusion-high-performance liquid chromatography (SEC-HPLC) analysis showed a single peak corresponding to purified *a*-PDL1-TGFβRII with no aggregation (Fig. 6c).

The comparative ability of *a*-PDL1-TGFβRII and *a*-PD-L1 to bind PD-L1 was evaluated by ELISA assay, wherein biotinylated recombinant human PD-L1 (rh B7-H1-biotin) was added to plates coated with *a*-PDL1-TGFβRII or *a*-PD-L1 antibody, and detected by horseradish peroxidase (HRP)-Avidin. Plates coated with nonspecific IgG-TGFβRII showed no binding to PD-L1 and served as a negative control. Each *a*-PDL1-TGFβRII (Ab1 and Ab2) exhibited specific binding to rhPD-L1 with an efficiency that was similar to the respective *a*-PD-L1 (Fig. 6d). The comparative ability of *a*-PDL1-TGFβRII and *a*-PD-L1 to bind TGFβ was evaluated by ELISA assay, wherein rhTGFβ1 was added to plates coated with *a*-PDL1-TGFβRII or *a*-PD-L1 and then detected by biotinylated anti-TGFβ1 and HRP-Avidin. Plates coated with nonspecific IgG-TGFβRII and rhTGFβRII-Fc served as positive controls to analyze the binding ability of the test samples to TGFβ. In contrast to the respective *a*-PD-L1 that failed to bind TGFβ, each corresponding *a*-PDL1-TGFβRII exhibited binding to TGFβ with an efficiency that was similar to the positive controls (Fig. 6e). The ability of *a*-PDL1-TGFβRII to simultaneously bind both PD-L1 and TGFβ was also evaluated by a bispecific ELISA assay, wherein *a*-PDL1-TGFβRII or *a*-PD-L1 was added to PD-L1-Fc-coated plates, followed by rhTGFβ1 that was detected by a biotinylated anti-human TGFβ1 antibody. In contrast to the respective *a*-PD-L1, each corresponding *a*-PDL1-TGFβRII (Ab1 and Ab2) exhibited simultaneous binding to PD-L1 and TGFβ (Fig. 6f).

### *a*-PDL1-TGFβRII is more effective than *a*-PD-L1 antibodies

**Fig. 7:**
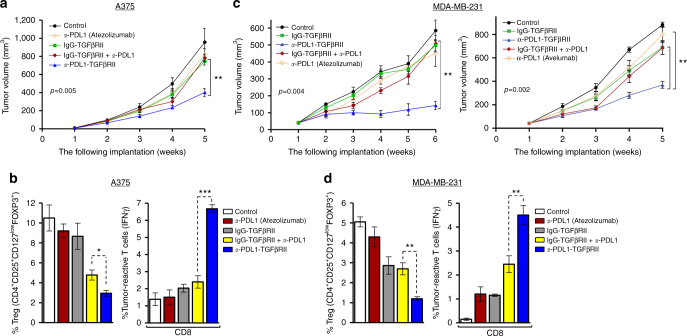
*a*-PDL1-TGFβRII exhibits superior antitumor efficacy compared to *a*-PD-L1 antibodies. Effect of the indicated treatments on melanoma and TNBC tumor xenografts in NSG mice reconstituted with human CD34^+^ HSC. Mice engrafted with human CD3^+^ cells were inoculated subcutaneously with either human melanoma (A375) or TNBC (MDA-MB-231-Luc) tumor cells. At 10d following tumor inoculation, mice were randomized into groups and treated i.p. with the indicated antibodies (5 mg kg^−1^ weekly) or vehicle alone (untreated control). Tumor growth curves in A375 tumor-bearing mice (**a**) or MDA-MB-231-Luc tumor-bearing mice (**c**) are shown (mean ± SEM of five to six mice in each indicated group). In TNBC model, independent experiments were conducted to compare two different *a*-PDL1-TGFβRII antibody–ligand traps with their respective *a*-PD-L1 antibodies, Atezolizumab (**c**, left) (and Avelumab (**c**, right). **b**, **d** Flow cytometric analysis of the comparative in vivo effect of the indicated treatments on infiltrating FOXP3-expressing Tregs and tumor-reactive IFN-γ-expressing CD8^+^ T cells in melanoma (**b**) and TNBC (**d**) models

The comparative antitumor efficacy of *a*-PDL1-TGFβRII, *a*-PD-L1, nonspecific IgG-TGFβRII, and the combination of *a*-PD-L1 and IgG-TGFβRII against human cancers expressing both PD-L1 and TGFβ was evaluated in either A375 (Fig. 7a, b) or MDA-MB-231-Luc (Fig. 7c, d) bearing NSG mice reconstituted with human CD34^+^ hematopoietic stem cells (HSCs). Moreover, in the TNBC model, independent experiments were conducted to compare two different *a*-PDL1-TGFβRII antibody-ligand traps with their respective *a*-PD-L1 antibodies (Atezolizumab and Avelumab) (Fig. 7c). In vivo tumor growth curves (mean ± SEM) in both tumor models demonstrated that treatment of tumor-bearing mice with *a*-PDL1-TGFβRII was significantly more effective at inhibiting the progression of A375 (*p* < 0.01, Student’s unpaired *t*-test) (Fig. 7a) or MDA-MB-231-luc (*p* < 0.004, Student’s unpaired *t*-test) (Fig. 7c) tumors compared with the respective *a*-PD-L1 alone, IgG-TGFβRII alone, and the combination of *a*-PD-L1 and nonspecific IgG-TGFβRII. Consistent with its superior antitumor efficacy, treatment with *a*-PDL1-TGFβRII resulted in significant inhibition of FOXP3^+^ expressing Tregs (*p* < 0.05, Student’s unpaired *t*-test) (Fig. 7b, d: left) and a greater elevation in percentage of tumor-reactive IFNγ-expressing CD8^+^ cells (*p* < 0.01, Student’s unpaired *t*-test) (Fig. 7b, d: right) compared with treatment with the *a*-PD-L1 alone, IgG-TGFβRII alone, and even their combination. Mice treated with *a*-PDL1-TGFβRII maintained serum hepatic enzymes within a normal range of liver function and demonstrated no loss of body weight during the course of experiment.

## Discussion

Cancer immunotherapy is currently focused on targeting immune inhibitory checkpoints that control T cell activation, such as CTLA-4 and PD-1^40,42,43,65–67^. Monoclonal antibodies that block these immune checkpoints can unleash antitumor immunity and produce durable clinical responses in a subset of patients with advanced cancers, such as melanoma and non-small-cell lung cancer^42,43,67^. However, these immunotherapeutics are currently constrained by their inability to induce clinical responses in the vast majority of patients. A key limitation of checkpoint inhibitors is that they narrowly focus on modulating the immune synapse but do not address the key molecular determinants that are primarily responsible for immune dysfunction in the tumor microenvironment^3,6–8,10,54^. Our data indicate that elevated expression of TGFβ is a root cause of such T-cell dysfunction in the tumor microenvironment. We find that autocrine and paracrine TGFβ signaling fundamentally affects tumor-infiltrating T cells by skewing the differentiation of T_H_1 cells toward a Treg phenotype, attenuating the activation of CD8^+^ effector cells and limiting the development of central memory cells. As Tregs express and employ TGFβ to maintain their own phenotype and function^13,18,19,44,59–61,68^, counteracting these deleterious cells and restoring beneficial T_H_1 cells is contingent upon making them impervious to such autocrine signaling. This poses the therapeutic challenge of specifically breaking this TGFβ-driven autocrine loop in tumor-infiltrating Tregs. Systemic TGFβ antagonists fall short of interrupting autocrine signaling in Tregs, as they lack preferential localization to T cells and fail to efficiently compete with the native TGFβRII receptor for binding TGFβ.

CTLA-4 antibodies, such as ipilimumab, can target FOXP3-expressing Tregs and counteract CTLA-4-mediated inhibition of B7-CD28 interaction^36^. However, *a*-CTLA-4 fails to counteract autocrine/paracrine TGFβ signaling, thereby resulting in NFAT/SMAD3-mediated upregulation of FOXP3^19^ and a paradoxical increase in tumor-infiltrating Tregs in the TGFβ-enriched immune microenvironment found in the majority of cancers^45^. Our data demonstrate that *a*-CTLA4-TGFβRII effectively addresses this challenge by exploiting the FOXP3-driven expression of CTLA-4 to not only disable the CTLA-4 checkpoint, but also decorate the targeted Tregs with a decoy TGFβRII ectodomain that traps TGFβ at the surface of the T cell, thereby rendering them virtually unresponsive to autocrine or paracrine TGFβ in their immediate milieu. As a result, *a*-CTLA4-TGFβRII counteracts autocrine/paracrine TGFβ/SMAD3-dependent expression of FOXP3, thereby reducing the differentiation and suppressive activity of Tregs. By skewing CD4^+^ T cells away from FOXP3^+^ Tregs or T_H_17 cells to a T_H_1-helper phenotype, *a*-CTLA4-TGFβRII enables effective activation of antitumor CD8^+^ effector T cells. An especially attractive feature of *a*-CTLA4-TGFβRII is its targeted ability to trap TGFβ at the surface of the T cell in a CTLA-4-directed manner, thereby interrupting the TGFβ-autocrine loop that drives FOXP3-mediated expression of CTLA-4. This distinguishing feature allows *a*-CTLA4-TGFβRII to enjoy a better therapeutic index compared with non-targeted TGFβ antagonists or even combinatorial therapy with a CTLA-4 antibody and a systemic TGFβ antagonist that is not directed to the T cell microenvironment. This unique ability to counteract Tregs and correct immune tolerance in a TGFβ-enriched tumor immune microenvironment enables *a*-CTLA4-TGFβRII to be significantly more effective in activating antitumor immunity and inhibiting tumor progression compared with a CTLA-4 antibody, a PD-1 antibody, or even the combination of both mAbs. Interestingly, *a*-CTLA4-TGFβRII was able to exhibit superior single agent activity against PD-L1-expressing tumors and the addition of *a*-PD1 antibody did not significantly enhance its antitumor efficacy in the breast cancer model. Although the highly effective counteraction of Tregs and immune tolerance by *a*-CTLA4-TGFβRII was sufficient to inhibit tumor growth, this might have obscured any potential value of combination therapy with *a*-PD1 during the course of the experiment. As IFN-γ-mediated upregulation of PD-L1 has been shown to be a mechanism of adaptive immune tolerance^39,69^, it remains possible that PD-1/PD-L1 blockade could potentially enhance the antitumor activity of *a*-CTLA4-TGFβRII over a more extended treatment period or in other tumor models.

Whereas CTLA-4 is highly expressed on Tregs, PD-L1 is overexpressed on tumor cells as well as tumor-infiltrating T cells, where it cooperates with TGFβ to inhibit T-cell activation and induce and maintain Tregs^70^. As PD-L1 and TGFβ entrain independent but cooperative mechanisms of immune tolerance, autocrine and paracrine TGFβ signaling in the tumor immune microenvironment may also limit the therapeutic efficacy of PD-1/PD-L1 antagonists. Consistent with this notion, our data demonstrate that *a*-PDL1-TGFβRII is significantly more effective in inhibiting tumor progression compared with the corresponding *a*-PD-L1 antibody due to its bifunctional ability to not only block PD-L1/PD-1 interaction, but simultaneously interrupt autocrine/paracrine TGF-β signaling in the localized microenvironment of PD-L1 expressing tumor-infiltrating immune cells and tumor cells.

Although humanized NSG mice used in this study exhibit a functionally validated surrogate human immune system^71^, this model supports the growth of human cancer cell line and PDXs even when they are not specifically HLA matched to the human CD34^+^ HSC used for immune reconstitution^72^. The absence of tumor rejection or inhibition of tumor progression in this model demonstrates that there is no spontaneous anti-allogeneic or tumor-specific immune response against such xenografts. However, as HLA-A*02 is the most highly prevalent HLA-A allele in patients with melanoma and breast cancer (including tumors cells used in this study), NSG mice were reconstituted with HLA-A*02 CD34^+^ HSCs. This was designed to ensure that HLA-A2-restricted TILs recognize HLA-A2-expressing xenografts, enabling generation of HLA-A2-restricted cytotoxic T cells. As such, our models were used to assess the comparative ability *a*-CTLA4-TGFβRII or *a*-PDL1-TGFβRII and their respective parent antibodies (*a*-CTLA-4 and *a*-PD-L1) to counteract immune tolerance in the TME and activate antitumor immune responses. Although these include HLA-A2-restricted responses against tumor antigens, they could exclude HLA-restricted T-cell responses to antigens presented by other class I or class II HLA loci that may not be matched. The elicited immune responses may not be restricted to tumor antigens, but also potentially encompass anti-allogeneic responses. As such, our tumor models stringently compare T-cell-mediated antitumor immune responses between treatment groups and controls under the same conditions, rather than estimate the absolute efficacy of each independent treatment.

Our preclinical studies indicate that both antibody-ligand traps (*a*-CTLA4-TGFβRII and *a*-PDL1-TGFβRII) have a superior therapeutic index compared with their parent immune checkpoint inhibitors that are currently in clinical use. Although no adverse events were observed in mice treated with either *a*-CTLA4-TGFβRII or *a*-PDL1-TGFβRII, any novel immunotherapeutic strategy that seeks to counteract Treg cells and unleash antitumor immunity carries a potential risk of autoimmune sequelae in patients. As such, the clinical translation of this approach requires well-designed phase I dose-escalation trials to carefully evaluate the safety of each novel agent, determine the maximum tolerated dose, and identify the optimal therapeutic dose and schedule that can elicit an antitumor immune response without prohibitive immune-related adverse events. As elevated TGFβ is an especially common denominator of immune dysfunction in many types of cancer, these Y-traps may provide an effective immunotherapeutic strategy against cancers that fail to respond to current immune checkpoint inhibitors by simultaneously disabling immune checkpoints and counteracting TGFβ-mediated immune tolerance.

## Methods

### Correlative analysis of TGFβ pathway and FOXP3 expression

RNA sequencing (RNA-Seq) data for 472 melanomas and 776 breast tumors were retrieved from TCGA. As unlike the breast cohort, TCGA melanoma collection lacks data from the healthy individuals, we have carefully selected a tissue specific normal control cohort (accession number GSE85861) form NCBI GEO repository. RNA-Seq data preprocessing and normalization steps were performed in R version 3.1.0 using DEseq package from Bioconductor. To adjust for the possible batch and processing effect, we have employed the XPN algorithm (R package, CONOR)^73^. The resulting matrix contained mRNA expression information for over 20K genes across all analyzed samples. Normalized gene expression data were loaded into iPANDA^50,51^. The software enables calculation of the Pathway Activation Score (PAS) for each of the 374 pathways analyzed, a value that serves as a quantitative measure of differential pathway activation. A collection of 374 intracellular signaling pathways (which cover a total of 2,294 unique genes) strongly implicated with various solid malignancies was obtained from the SABiosciences (http://www.sabiosciences.com/pathwaycentral.php), and used for the computational algorithm as described previously^50,51^. Calculated PAS values for TGFβ pathway were used for correlative analysis with *TGFB1*, *FOXP3*, and *PDL1* expression levels seen in the same patients.

### Design of *a*-CTLA4-TGFβRII and *a*-PDL1-TGFβRII

Anti-CTLA4-TGFβRII was designed by fusing the C terminus of the HC of a human anti-CTLA-4 antibody (Ipilimumab) with a ligand-binding sequence of the extracellular domain of TGFβRII (TGFβRII ECD) via a flexible linker peptide, (GGGGS)_3_. Anti-PDL1-TGFβRII was designed to simultaneously target both PD-L1 and TGF-β by fusing the C terminus of the HC of human anti-PD-L1 antibody (Atezolizumab and Avelumab) with a ligand-binding sequence of the extracellular domain of TGFβRII (TGFβRII ECD) via a flexible linker peptide, (GGGGS)_3_. The amino acid sequences were codon optimized with GeneOptimizer (Life Technologies). Anti-gp120-TGFβRII antibody was used as a non-specific IgG-TGFβRII control. Amino acid sequences of all fusion antibodies used in this study are provided in Supplementary Material. The complementary DNA for the antibody HC and the cDNA for the antibody LC were gene synthesized and subsequently cloned into separate plasmids (pEvi3; evitria AG, Switzerland) under the control of a mammalian promoter and polyadenylation signal. Plasmid DNA was amplified in *Escherichia coli* and DNA was purified using anion exchange kits for low endotoxin plasmid DNA preparation. The plasmid DNAs for HC and LC were subsequently co-transfected into CHO K1 cells with eviFect (evitria AG, Switzerland), and the CHO cells were cultured in eviMake (evitria AG, Switzerland), a serum-free, animal-component-free medium. Production was terminated once viability reached 75%, which occurred at day 8 after transfection. The antibody-containing supernatant was then harvested and antibody was purified at 20 ^o^C by Protein A affinity chromatography on a Bio-Rad BioLogic FuoFlow FPLC machine with subsequent gel filtration as polishing and re-buffering step. The purified antibody was re-buffered into phosphate-buffered saline, sterile-filtered, aliquoted, and frozen at − 80 ^o^C. Protein identification of the purified antibody from CHO cell supernatants was performed by LC–MS/MS to confirm the amino acid sequence and size of the HC of *a*-CTLA4-TGFβRII and *a*-PDL1-TGFβRII (Mass Spectrometry and Proteomics Facility, Johns Hopkins University School of Medicine). SDS-PAGE under R and NR conditions was used to compare the FL, HC, and LC of *a*-CTLA4-TGFβRII with *a*-CTLA-4, and *a*-PDL1-TGFβRII with *a*-PD-L1.

### Bifunctional target-binding ability of fusion antibodies

The ability of anti-CTLA4-TGFβRII antibody to simultaneously bind both CTLA-4 and TGFβ was evaluated by a ‘double-sandwich’ ELISA, wherein anti-CTLA4-TGFβRII or anti-CTLA-4 antibody (1 μg ml^−1^) was added to CTLA-4-Fc-coated plates, followed by rhTGFβ1 (0–2,000 pg ml^−1^) that was detected by a biotinylated anti-human TGFβ1 antibody (R&D Systems). The positive standard curve (TGFβRII-Fc-coated plate) was used to analyze the binding ability of the test samples. The ability of *a*-CTLA4-TGFβRII to bind TGFβ1 was also evaluated by competition ELISA. The ELISA plate was coated with the capture antibody (*a*-TGFβ, 1 μg ml^−1^), followed by rhTGFβ1 in the presence of either *a*-CTLA4-TGFβRII or *a*-CTLA-4 (Antibody : TGFβ1 ratio 1 : 1 to 100 : 1) for 1 h at room temperature. Each experiment was performed twice, with triplicate wells for each indicated condition.

The comparative ability of anti-PDL1-TGFβRII and anti-PD-L1 antibody to bind PD-L1 was evaluated by ELISA, wherein biotinylated recombinant human PD-L1 (rh B7-H1-biotin; 0–100 ng ml^−1^; R&D Systems) was added to plates coated with anti-PDL1-TGFβRII or anti-PD-L1 antibody (1 μg ml^−1^), followed by detection with HRP-Avidin. Plates coated with nonspecific IgG-TGFβRII served as a negative control to analyze the binding ability of the test samples. The comparative ability of *a*-PDL1-TGFβRII and *a*-PD-L1 antibody to bind TGFβ was evaluated by ELISA, wherein rhTGFβ1 (0–2,000 pg ml^−1^) was added to plates coated with *a*-PDL1-TGFβRII or *a*-PD-L1 antibody (1 μg ml^−1^), which was detected by biotinylated anti-TGFβ1 and HRP-Avidin (R&D Systems). Plates coated with nonspecific IgG-TGFβRII and rhTGFβRII-Fc served as positive controls to analyze the binding ability of the test samples to TGFβ. The ability of *a*-PDL1-TGFβRII to simultaneously bind both PD-L1 and TGF-β was evaluated by a bispecific ELISA, wherein *a*-PDL1-TGFβRII or *a*-PD-L1 antibody (0–100 ng ml^−1^) was added to PD-L1-Fc-coated plates (1 μg ml^−1^), followed by rhTGFβ1 (100 ng ml^−1^) that was detected by a biotinylated anti-human TGFβ1 antibody (R&D Systems).

### TGFβ-binding ability of *a*-TGFβ and IgG-TGFβRII

The ability of *a*-TGFβ (1D11) and nonspecific IgG-TGFβRII (anti-gp120-TGFβRII) antibody to equally bind TGFβ in vitro was evaluated by a standard ELISA assay (Supplementary Figure 1). rhTGFβ1 (0–2000 pg ml^−1^) was added to the plates coated with either TGFβRII-Fc (R&D Systems), *a*-TGFβ (Bioxcel), or IgG-TGFβRII (1 μg ml^−1^ each), and binding to rhTGFβ1 was detected by a biotinylated anti-human TGFβ1 antibody (R&D Systems). TGFβRII-Fc-coated plates were used as a TGFβ-binding positive control (Supplementary Figure 1). To demonstrate that both agents were administered at doses sufficient to saturate systemic TGFβ in vivo, sequestration of serum TGFβ was assessed in A375 tumor-bearing NSG immune-reconstituted mice treated with either *a*-TGFβ or IgG-TGFβRII (5 mg kg^−1^ per week) for 4 weeks (Supplementary Figure 2). At the endpoint, serum was collected from tail bleed and levels of TGFβ were detected using the TGFβ-1 Human ELISA Kit (ThermoFisher Scientific) following the manufacturer’s protocol.

### TGFβ-induced FOXP3 expression and Treg differentiation

Human PBMCs (ALLCELLS) were stained with anti-CD3 and Glycophorin A to enumerate T cells. Cells were cultured with rhIL-2 (100 IU ml^−1^) and anti-CD3/anti-CD28-coated Dynabeads (Life Technologies) at a ratio of 1 : 3 (cell : bead) in the presence or absence of 2.5 ng ml^−1^ rhTGF-β1 with or without either *a*-CTLA4-TGFβRII or *a*-CTLA-4 antibody (5 μg ml^−1^). Following culture for 24–48 h, cells were lysed and subjected to immunoblot analyses with the following primary antibodies: FOXP3, SMAD-2/3 (D7G7), or phospho-SMAD-2/3, and β-actin (Cell Signaling Technologies). On day 5, anti-CD3/anti-CD28 beads were magnetically removed and the number of Tregs (CD4^+^/CD25^high^/CD127^low^/FOXP3^+^) were enumerated by flow cytometry. Cells were stained extracellularly with anti-human CD4-PE, anti-human CD25-PE-CY^TM^5, and anti-human CD127-FITC antibodies (BD Biosciences). The cells were then permeabilized (BD Cytofix/Cytoperm Kit) and stained intracellularly with anti-human FOXP3-APC or the corresponding isotype control mouse IgG1-APC (eBioscience). The stained cells were washed twice with FACS buffer, run on the Gallios Flow Cytometer, and analyzed utilizing Kaluza Software (Beckman Coulter).

### Analysis of Treg suppressor function

Patient-derived tumor-infiltrated BM (myeloma-BM) was stained with anti-CD3 and Glycophorin A to enumerate T cells. Following activation for 7 days with rhIL-2 and anti-CD3/anti-CD28 beads, the cells were magnetically separated and stained with anti-CD3. Concurrently, autologous Tregs were isolated from the same patient’s peripheral blood lymphocytes using anti-CD4/anti-CD25 beads (Miltenyi Biotechnology). The activated T cells were CFSE labeled (Life Technologies) and added to autologous BM that had been pulsed for 30 min in medium with or without either tumor-specific antigen (tumor cell lysate) or nonspecific antigen (nonspecific cell lysate) and plated in the presence or absence of the selected autologous Tregs. Following culture for 3 days with or without either *a*-CTLA4-TGFβRII or *a*-CTLA-4 antibody (5 μg ml^−1^), the cells were stained with anti-CD3 and anti-IFNγ. Tumor antigen-specific T cells were considered as CD3^+^/CFSE^low^/IFNγ^+^.

### Analysis of T_H_17 cell differentiation

PBMCs were co-incubated with anti-CD3/CD28 beads (1 : 3 cell to bead ratio) in AIM-V medium (Invitrogen) in the presence of IL17-skewing cytokines (10 ng ml^−1^ IL-6, 5 ng ml^−1^ TGFβ, 10 ng ml^−1^ anti–IFN-γ, and 10 ng ml^−1^ anti–IL-4 (R&D Systems)) with either *a*-CTLA4-TGFβRII or *a*-CTLA-4 antibody (5 μg ml^−1^). Following incubation for 3 days, Leukocyte Activation Cocktail (BD Biosciences) was added at 2 μl ml^−1^ of cell culture for 4 h. Cells were collected and stained extracellularly with anti-human CD4 (BD Biosciences), permeabilized (BD Cytofix/Cytoperm Kit), and stained intracellularly with IL-17 and IFN-γ antibodies (eBioscience). The stained cells were washed twice with FACS buffer, run on the Gallios Flow Cytometer, and analyzed utilizing Kaluza Software (Beckman Coulter).

### Tumor cell lines and treatments

A375 and SK-MEL-5 human melanoma cell lines were purchased from ATCC and maintained according to ATCC guidelines. MDA-MB-231 is a metastatic human TNBC cell line with mesenchymal-like morphology (Basal B-like). MDA-MB-231-Luc (D3H2LN) is a TNBC subline with enhanced primary tumor growth and lung metastases that was derived from a metastatic deposit of bioluminescent MDA-MB-231 cells stably expressing firefly luciferase. MDA-MB-231 cells were maintained in Dulbecco’s modified Eagle’s medium supplemented with 10% fetal bovine serum and penicillin/streptomycin. All cell lines were periodically monitored for mycoplasma at Johns Hopkins Genetic Resources Core Facility using the MycoDtect kit (Greiner Bio-One) and authenticated using genetic fingerprinting (Identifiler, Applied Biosystems) before use.

### Treatment of NSG mice reconstituted with human CD34^+^ cells

Female immune-deficient NSG mice (NOD/Shi-*scid IL-2rg*^*null*^)(4–8-week old) were irradiated at 200 cGy, followed by adoptive transfer of human BM CD34^+^ cells (7 × 10^4^ per mouse) from a normal donor (HLA-matched to the tumor) (ALLCELLS). Mice were tested for engraftment of human CD3^+^ T cells in peripheral blood obtained via tail-bleed at 7–8 weeks following injection of CD34^+^ cells. The cells were stained with anti-CD3-APC and anti-CD19-FITC antibodies. Mice demonstrating engraftment of human CD3^+^ cells were inoculated with either human melanoma tumor cells (A375 or SK-MEL-5) (2 × 10^6^ cells subcutaneously), PDX (subcutaneously), or human TNBC cells (MDA-MB-231-Luc) (2 × 10^6^ in Matrigel; mammary fat pad). At 10d following tumor inoculation, mice were allocated to groups using blinded block randomization and treated (5 mg kg^−1^ intraperitoneally (i.p.) weekly) with either vehicle alone (untreated control) or the following antibodies: *a*-CTLA4-TGFβRII; *a*-CTLA-4 (ipilimumab); nonspecific IgG-TGFβRII (*a*-gp120-TGFβRII); *a*-TGFβ (1D11); *a*-PD-1 (pembrolizumab); combination of *a*-CTLA-4 and IgG-TGFβRII; combination of *a*-CTLA-4 and *a*-TGFβ; combination of *a*-CTLA4 and *a*-PD-1; combination of *a*-CTLA4-TGFβRII and *a*-PD-1; *a*-PDL1-TGFβRII (Ab1 and Ab2); *a*-PD-L1 (atezolizumab and avelumab); *a*-PD-L1 + IgG-TGFβRII; and Vehicle alone (Control). Tumor size was measured weekly blinded to the treatment group and tumor volume was calculated using the formula (length × width × height).

### Immunophenotype analysis of human tumor-bearing mice

Tumors and BM were collected from tumor-bearing mice in each group for immunophenotype analysis of T cells. Tumor samples were subjected to collagenase digestion for 20 min at 37 °C followed by red blood cell lysis. Tregs in tumor-infiltrating or BM T cells were measured by flow cytometric analysis of CD4^+^CD25^+^CD127^low^FOXP3^+^ cells, as described above. Tumor-infiltrating or BM T cells were stained with anti-human CD3-PE, anti-human CD8-PE-CY^TM^7, anti-human CD45RO-APC, and anti-human CD62L-FITC, and analyzed by flow cytometry to quantify T cells with a central memory phenotype (CD45RO^high^CD62L^high^). To evaluate tumor-specific IFN-γ expression in CD3^+^/CD8^+^ T cells, BM cells were plated in 96-well plates (2 × 10^**5**^ cells per well) in the presence of tumor cell lysate, nonspecific control peptide, or medium alone. Cells were cultured for 72 h followed by incubation with Golgi stop for 4 h. Cells were stained extracellularly with anti-CD3-FITC and anti-CD8-APC antibodies, permeabilized, stained intracellularly with anti-IFN-γ-PE or its corresponding isotype control, and then analyzed by flow cytometry. All the antibodies were from BD Biosciences.

### Bioluminescent imaging of primary and metastatic tumors

Tumor burden in mice bearing MDA-MB-231-Luc was assessed by visualization of in vivo luciferase activity using a Xenogen IVIS Spectrum system. Images were acquired at 10 min post injection of 50 mg kg^−1^ i.p. dose of luciferin. To detect metastases, the lower portion of each animal was shielded before re-imaging to minimize bioluminescence from the primary tumor. Lungs were harvested and imaged ex vivo to confirm in vivo observations. Photon flux was used to quantify the differences in tumor burden between treatment groups.

### Immunoblot analysis

RIPA buffer (Cell Signaling) was used for cell lysis according to manufacturer’s instructions and the protein concentrations were determined by BCA Protein Assay (Thermo Scientific). Proteins were separated by Criterion SDS-PAGE using Bis-Tris 4–12% gradient polyacrylamide gels (BioRad) and transferred to polyvinylidene difluoride membranes according to standard protocols. The primary antibodies were from Cell Signaling Technology: FOXP3 (Cat. 5298), phospho-SMAD2/3 (Cat. 3101), total SMAD2/3 (Cat. 3102), and β-actin (Cat. 8457). Secondary antibodies were from GE Healthcare: anti-rabbit IgG (NA914) and anti-mouse IgG (NA931). The uncropped scans are provided in Supplementary Figures 3–5.

### Human cell and tissue samples

Approval for research on human subjects was obtained from The Johns Hopkins University Institutional Review Board. This study qualified for exemption under the U.S. Department of Health and Human Services policy for protection of human subjects (45 CFR 46.101(b)) (IRB 03-11-12-06e). BM, peripheral blood, and tumor samples were obtained from patients and normal donors under informed consent in accordance with the Declaration of Helsinki with approval from the Institutional Review Board at Johns Hopkins University. Plasma was removed by centrifugation and stored at − 80 °C. Lymphocytes were obtained by Ficoll-Hypaque density gradient centrifugation (GE Healthcare).

### Animal use and care

The animals were maintained in accordance with guidelines of the American Association of Laboratory Animal Care and a research protocol approved by the Johns Hopkins University Animal Use and Care Committee.

### Statistical analyses and interpretation

All data are presented as the mean ± SEM. Student’s unpaired *t*-test (two-sided) was used to analyze differences between two groups. When appropriate, the Bonferroni correction was applied to account for multiple comparisons. Results with *p* < 0.05 were considered significant. Statistical analyses were conducted using GraphPad InStat (GraphPad Software). *P-*values are summarized as: *P ≤ 0.05, **P ≤ 0.01, and ***P ≤ 0.001, unless stated otherwise.

### Data availability

The TCGA data referenced in the study are available in a public repository from the National Cancer Institute Cancer Genome Atlas website. All the other data supporting the findings of this study are available within the article and its supplementary information files.

## Electronic supplementary material

Supplementary Information
