# Supplementary material for: Bifunctional immune checkpoint-targeted antibody-ligand traps that simultaneously disable TGFβ enhance the efficacy of cancer immunotherapy
Source: Nat Commun. 2018 Feb 21;9:741. doi: 10.1038/s41467-017-02696-6 (PMC5821872; doi:10.1038/s41467-017-02696-6)
Supplement: Supplementary file 1 — Supplementary Information [file 41467_2017_2696_MOESM1_ESM.pdf]

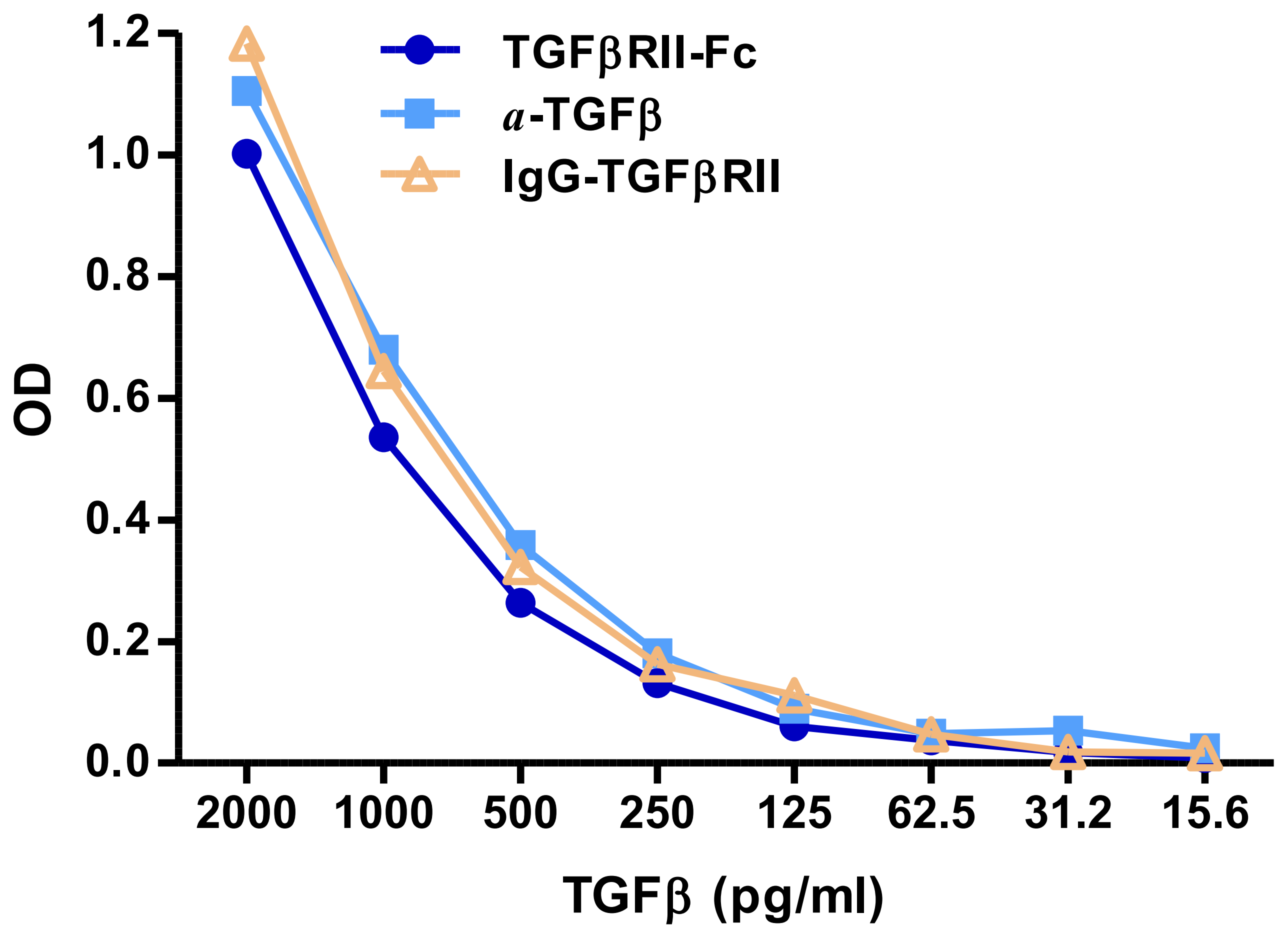


**Supplementary Figure 1. Comparative ability of *a*-TGFβ and IgG-TGFβRII to bind TGFβ *in vitro*.** Standard ELISA assay was performed to evaluate the comparative ability of *a*-TGFβ Ab (1D11) and IgG-TGFβRII to bind TGFβ *in vitro*. rhTGFβ1 (0-2000 pg/ml) was added to the plates coated with either TGFβRII-Fc, IgG-TGFβRII or *a*-TGFβ and binding to TGFβ was detected by a biotinylated anti-human TGFβ1 antibody. TGFβRII-Fc coated plates were used as a TGFβ-binding positive control. The data shows optical density (OD) values (mean of three replicate wells for each assay condition) from a representative of three independent experiments.

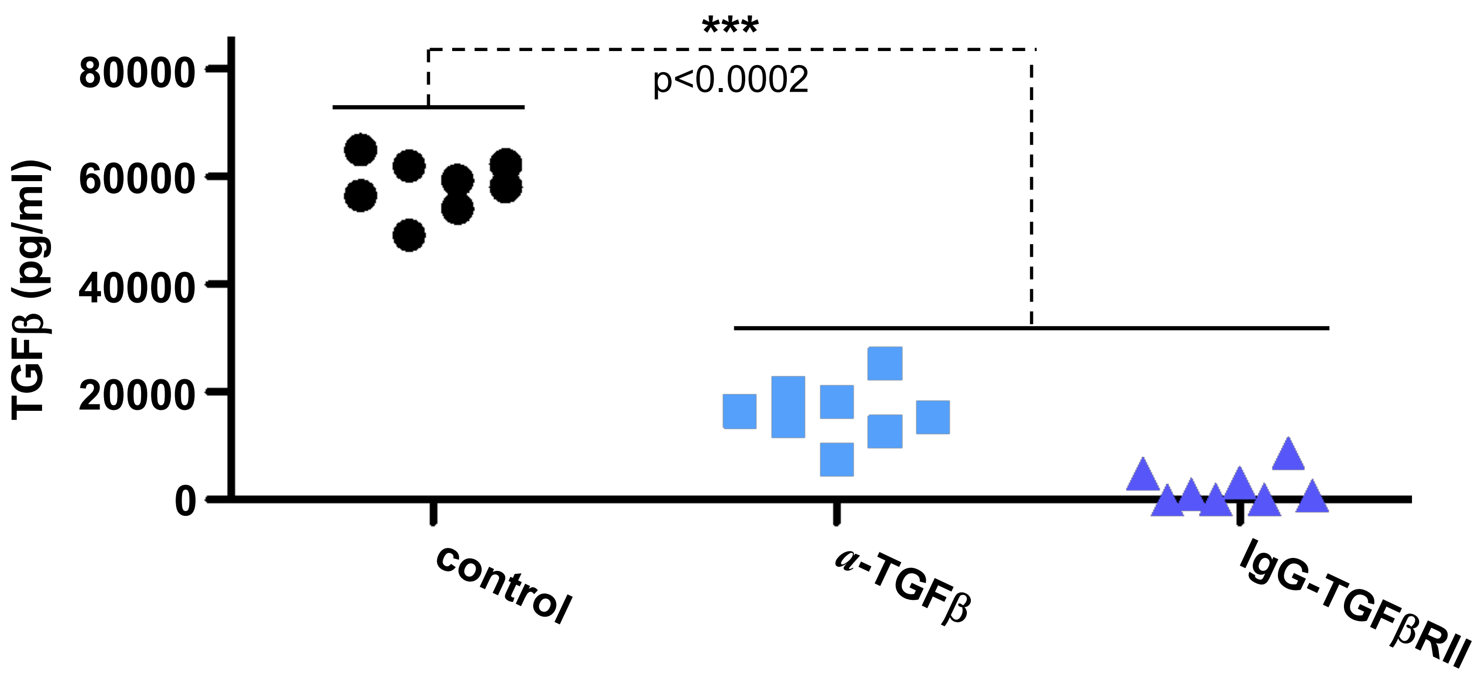


**Supplementary Figure 2. Sequestration of serum TGFβ by *a*-TGFβ and IgG-TGFβRII.** To demonstrate that both agents were administered at doses sufficient to saturate TGFβ *in vivo*, the sequestration of serum TGFβ was assessed in A375 tumor bearing mice treated with either *a*-TGFβ and IgG-TGFβRII at the dose used in all our *in vivo* studies (5mg/kg/week). Serum TGFβ levels in untreated tumor-bearing mice was significantly higher than that detected in cohorts treated with each antibody ($p<0.0002$).

# Uncropped scans - Figure 2c

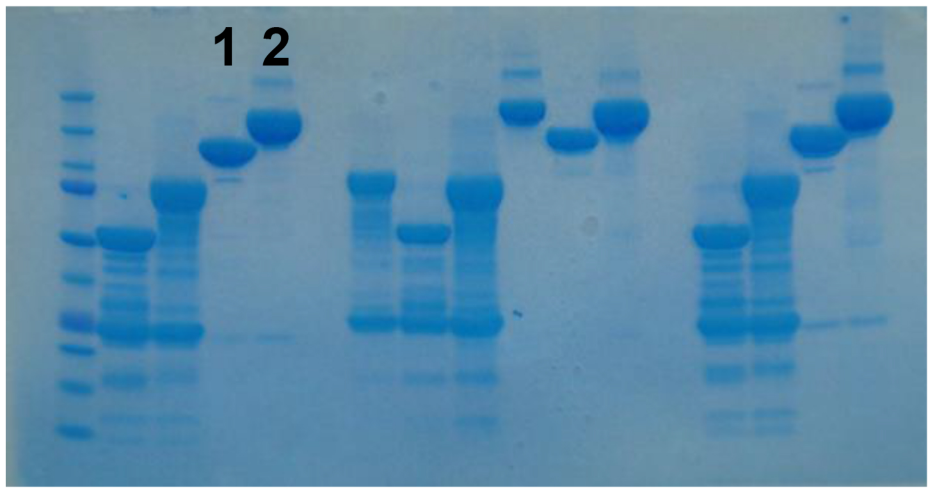


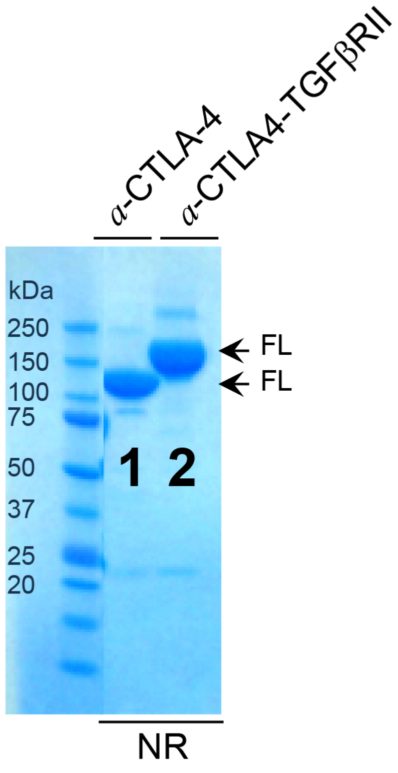


**Supplementary Figure 3.**

# Uncropped scans - Figure 3a

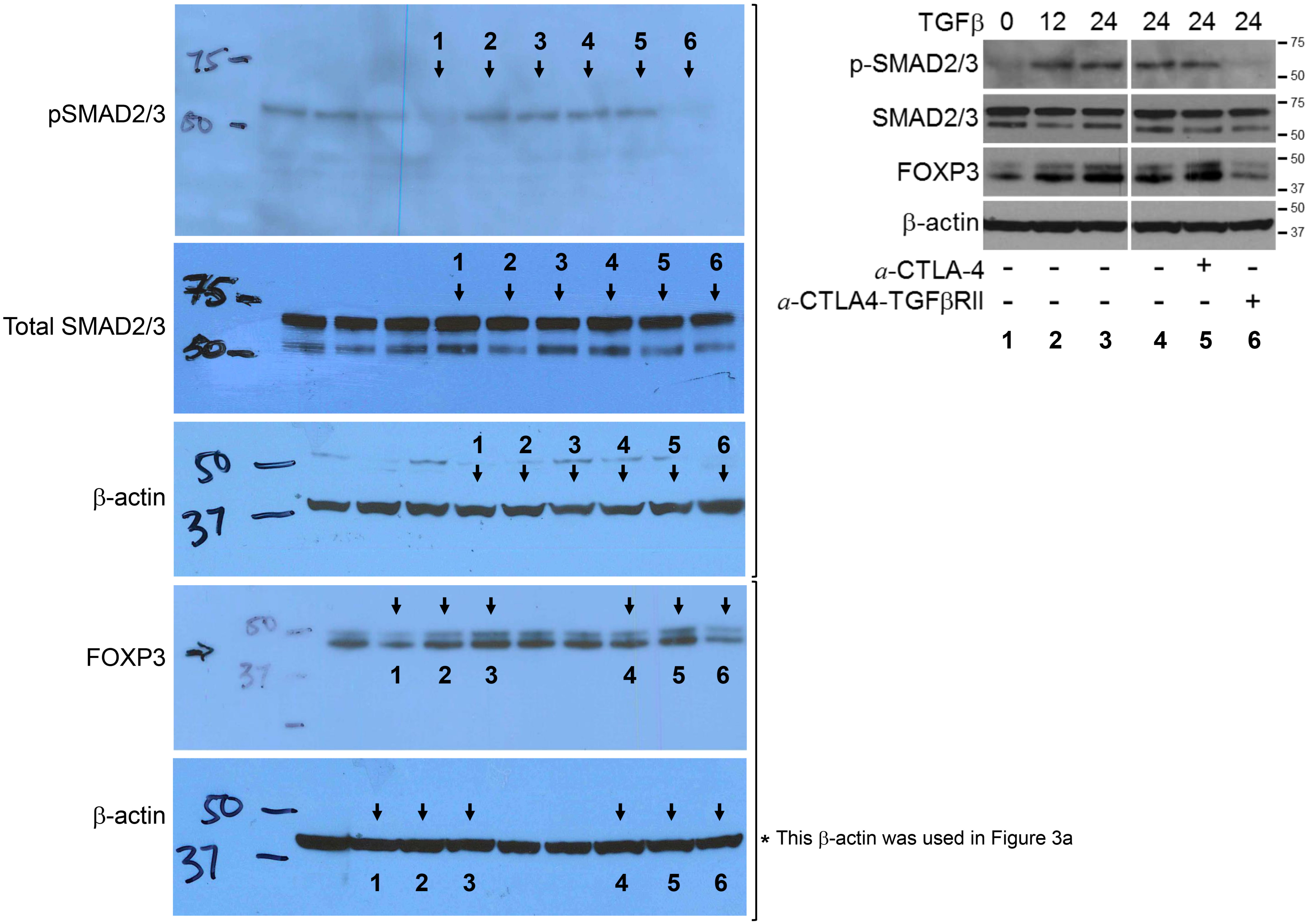


**Supplementary Figure 4.**

# Uncropped scans - Figure 6b

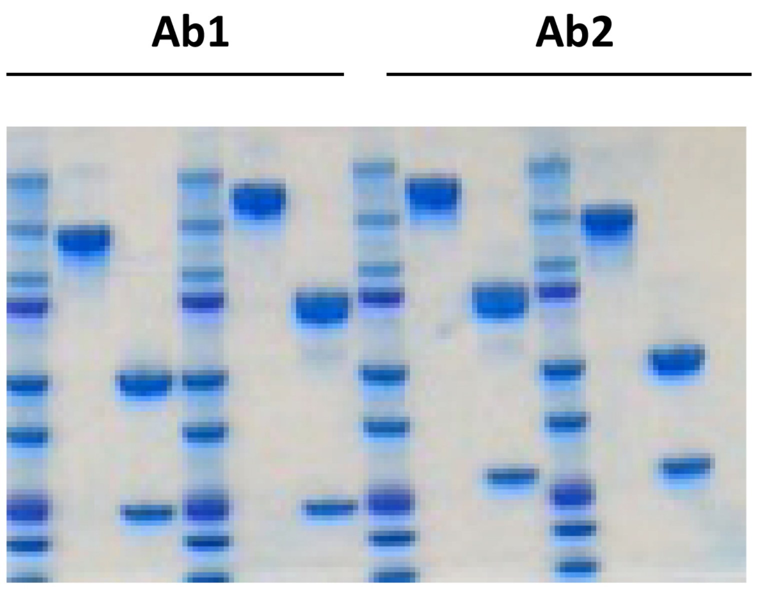


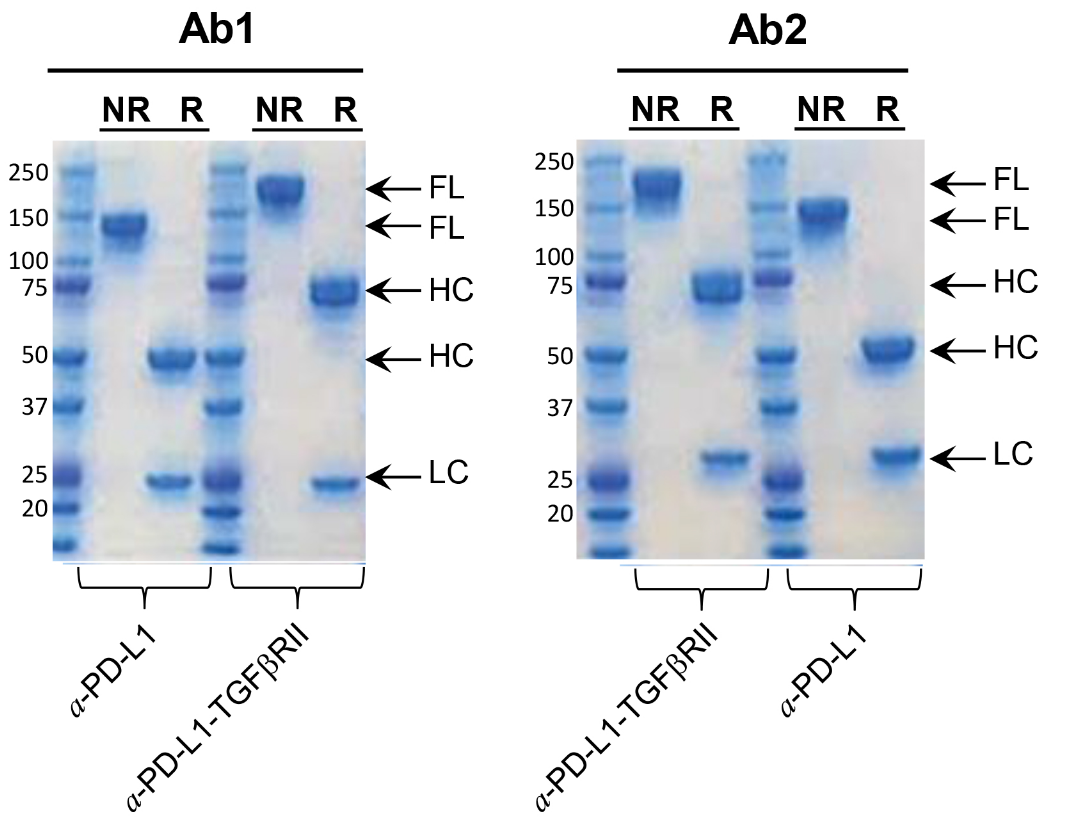


## Supplementary Figure 5.

## Supplementary Note 1

### Amino acid sequences of all fusion antibodies used in this study

**Anti-CTLA4-TGFβRII (Ipilimumab-TGFβRII)**

*Heavy chain*

QVQLVESGGGVVQPGRSLRLSCAASGFTFSSYTMHWVRQAPGKGLEWVTFISYDGNNKYYADSVKGRFTISRD
NSKNTLYLQMNSLRAEDTAIYYCARTGWLGPFDYWGQGTLVTVSSASTKGPSVFPLAPSSKSTSGGTAALGCLV
KDYFPEPVTVSWNSGALTSGVHTFPAVLQSSGLYSLSSVVTVPSSSLGTQTYICNVNHKPSNTKVDKRVEPKSCD
KTHTCPPCPAPELLGGPSVFLFPPKPKDTLMISRTPEVTCVVVDVSHEDPEVKFNWYVDGVEVHNAKTKPREEQ
YNSTYRVVSVLTVLHQDWLNGKEYKCKVSNKALPAPIEKTISKAKGQPREPQVYTLPPSRDELTKNQVSLTCLV
KGFYPSDIAVEWESNGQPENNYKTTPPVLDSDGSFFLYSKLTVDKSRWQQGNVFSCSVMHEALHNHYTQKSLSL
SPGKGGGGSGGGGSGGGGSTIPPHVQKSVNNDMIVTDNNGAVKFPQLCKFCDVRFSTCDNQKSCMSNCSITSICE
KPQEVCVAVWRKNDENITLETVCHDPKLPYHDFILEDAASPKCIMKEKKKPGETFFMCSCSSDECNDNIIFSEEY
NTSNPD

*Light chain*

EIVLTQSPGTLSLSPGERATLSCRASQSVGSSYLAWYQQKPGQAPRLLIYGAFSRATGIPDRFSGSGSGTDFTLTIS
RLEPEDFAVYYCQQYGSSPWTFGQGTKVEIKRTVAAPSVFIFPPSDEQLKSGTASVVCLLNNFYPREAKVQWKV
DNALQSGNSQESVTEQDSKDSTYSLSSTLTLSKADYEKHKVYACEVTHQGLSSPVTKSFNRGEC

**Anti-PDL1-TGFβRII (Atezolizumab-TGFβRII)**

*Heavy chain*

EVQLVESGGGLVQPGGSLRLSCAASGFTFSDSWIHWVRQAPGKGLEWVAWISPYGGSTYYADSVKGRFTISADT
SKNTAYLQMNSLRAEDTAVYYCARRHWPGGFDYWGQGTLVTVSAASTKGPSVFPLAPSSKSTSGGTAALGCLV
KDYFPEPVTVSWNSGALTSGVHTFPAVLQSSGLYSLSSVVTVPSSSLGTQTYICNVNHKPSNTKVDKKVEPKSCD
KTHTCPPCPAPELLGGPSVFLFPPKPKDTLMISRTPEVTCVVVDVSHEDPEVKFNWYVDGVEVHNAKTKPREEQ
YASTYRVVSVLTVLHQDWLNGKEYKCKVSNKALPAPIEKTISKAKGQPREPQVYTLPPSREEMTKNQVSLTCLV
KGFYPSDIAVEWESNGQPENNYKTTPPVLDSDGSFFLYSKLTVDKSRWQQGNVFSCSVMHEALHNHYTQKSLSL
SPGKGGGGSGGGGSGGGGSTIPPHVQKSVNNDMIVTDNNGAVKFPQLCKFCDVRFSTCDNQKSCMSNCSITSICE
KPQEVCVAVWRKNDENITLETVCHDPKLPYHDFILEDAASPKCIMKEKKKPGETFFMCSCSSDECNDNIIFSEEY
NTSNPD

*Light chain*

DIQMTQSPSSLSASVGDRVTITCRASQDVSTAVAWYQQKPGKAPKLLIYSASFLYSGVPSRFSGSGSGTDFTLTISS
LQPEDFATYYCQQYLYHPATFGQGTKVEIKRTVAAPSVFIFPPSDEQLKSGTASVVCLLNNFYPREAKVQWKVD
NALQSGNSQESVTEQDSKDSTYSLSSTLTLSKADYEKHKVYACEVTHQGLSSPVTKSFNRGEC

**Anti-PDL1-TGFβRII (Avelumab-TGFβRII)**

*Heavy chain*

EVQLLESGGGLVQPGGSLRLSCAASGFTFSSYIMMWVRQAPGKGLEWVSSIYPSGGITFYADTVKGRFTISRDNS
KNTLYLQMNSLRAEDTAVYYCARIKLGTVTTVDYWGQGTLVTVSSASTKGPSVFPLAPSSKSTSGGTAALGCLV
KDYFPEPVTVSWNSGALTSGVHTFPAVLQSSGLYSLSSVVTVPSSSLGTQTYICNVNHKPSNTKVDKRVEPKSCD
KTHTCPPCPAPELLGGPSVFLFPPKPKDTLMISRTPEVTCVVVDVSHEDPEVKFNWYVDGVEVHNAKTKPREEQ
YNSTYRVVSVLTVLHQDWLNGKEYKCKVSNKALPAPIEKTISKAKGQPREPQVYTLPPSREEMTKNQVSLTCLV
KGFYPSDIAVEWESNGQPENNYKTTPPVLDSDGSFFLYSKLTVDKSRWQQGNVFSCSVMHEALHNHYTQKSLSL
SPGKGGGGSGGGGSGGGGSTIPPHVQKSVNNDMIVTDNNGAVKFPQLCKFCDVRFSTCDNQKSCMSNCSITSICE
KPQEVCVAVWRKNDENITLETVCHDPKLPYHDFILEDAASPKCIMKEKKKPGETFFMCSCSSDECNDNIIFSEEY
NTSNPD

*Light chain*

QSALTQPASVSGSPGQSITISCTGTSSDVGGYNYVSWYQQHPGKAPKLMIYDVSNRPSGVSNRFSGSKSGNTASL
TISGLQAEDEADYYCSSYTSSSTRVFGTGTKVTVLGQPKANPTVTLFPPSSEELQANKATLVCLISDFYPGAVTVA
WKADGSPVKAGVETTKPSKQSNNKYAASSYLSLTPEQWKSHRSYSCQVTHEGSTVEKTVAPTECS

**<u>Anti-gp120-TGFβRII (B12-TGFβRII) – control antibody</u>**

*Heavy chain*

QVQLVQSGAEVKKPGASVKVSCQASGYRFSNFVIHWVRQAPGQRFEWMGWINPYNGNKEFSAKFQDRVTFTA
DTSANTAYMELRSLRSADTAVYYCARVGPYSWDDSPQDNYYMDVWGKGTTVIVSSASTKGPSVFPLAPSSKST
SGGTAALGCLVKDYFPEPVTVSWNSGALTSGVHTFPAVLQSSGLYSLSSVVTVPSSSLGTQTYICNVNHKPSNTK
VDKRVEPKSCDKTHTCPPCPAPELLGGPSVFLFPPKPKDTLMISRTPEVTCVVVDVSHEDPEVKFNWYVDGVEV
HNAKTKPREEQYNSTYRVVSVLTVLHQDWLNGKEYKCKVSNKALPAPIEKTISKAKGQPREPQVYTLPPSREEM
TKNQVSLTCLVKGFYSDIAVEWESNGQPENNYKTTPPVLDSDGSFFLYSKLTVDKSRWQQGNVFSCSVMHEAL
HNHYTQKSLSLSPGKGGGGSGGGGSGGGGSTIPPHVQKSVNNDMIVTDNNGAVKFPQLCKFCDVRFSTCDNQK
SCMSNCSITSICEKPQEVCVAVWRKNDENITLETVCHDPKLPYHDFILEDAASPKCIMKEKKKPGETFFMCSCSSD
ECNDNIIFSEEYNTSNPD

*Light chain*

EIVLTQSPGTLSLSPGERATFSCRSSHSIRSRRVAWYQHKPGQAPRLVIHGVSNRASGISDRFSGSGSGTDFTLTITR
VEPEDFALYYCQVYGASSYTFGQGTKLERKRTVAAPSVFIFPPSDEQLKSGTASVVCLLNNFYPREAKVQWKVD
NALQSGNSQESVTEQDSKDSTYSLSSTLTLSKADYEKHKVYACEVTHQGLSSPVTKSFNRGEC
